# Perinatal health among migrant women: A longitudinal register study in Finland 2000-17

**DOI:** 10.1016/j.ssmph.2022.101298

**Published:** 2022-11-24

**Authors:** Heini Väisänen, Hanna Remes, Pekka Martikainen

**Affiliations:** a Institut National d’Etudes Démographiques (INED), Aubervilliers, France; b Department of Social Statistics and Demography, University of Southampton, Southampton, UK; c Population Research Unit, Faculty of Social Sciences, University of Helsinki, Helsinki, Finland; d Laboratory of Population Health, Max Planck Institute for Demographic Research, Rostock, Germany

**Keywords:** Reproductive health, Migrant health, Healthy immigrant effect, Weathering hypothesis, Finland, Register data

## Abstract

Migrants often have better health than the native-born population (‘healthy immigrant effect’), although the effect tends to attenuate over time since migration. However, following the weathering hypothesis, migrants may have worse health due to a combination of discrimination and poorer financial conditions faced by many of them. Yet, little is known about interactions between migrant origin and individual socioeconomic status or the time spent in the host country in relation to reproductive health. We use Finnish register data of 491,532 women and 948,616 births spanning years 2000-17 to longitudinally study the association between the country of birth and perinatal outcomes (preterm birth, unplanned C-section, episiotomy and spontaneous vaginal birth); the interaction of country of birth with household income; and the effect of time since migration using random intercept logistic regression models. We show that a ‘healthy immigrant effect’ largely does not exist for perinatal outcomes apart from migrants from a few high-income countries. Instead, in particular women from poorer countries tended to fare worse than native women. Often, the effect of the country of birth did not differ by household income, or the patterns were not clear. The impact of time since immigration was complex and dependent on country of birth and the outcome studied, but showed an increase in risk of preterm birth among migrants from low- and lower-middle-income countries compared to those born in Finland. Discrimination, language barriers in seeking care or refugee experiences are among some of the possible mechanisms explaining the worse perinatal health of migrants from poorer countries. The inequalities observed in a global scale in countries' economic outcomes may reproduce themselves as reproductive health inequalities among migrants living in wealthy countries.

## Highlights

• We found no ‘healthy immigrant effect’ for perinatal outcomes apart from migrants from a few high-income countries.
• Household income mostly did not explain or interact with the effect of the country of birth on reproductive health.
• Time since migration had a positive association with preterm birth risk for low and lower middle income country migrants.
• Global inequalities may reproduce themselves as reproductive health inequalities among migrants in wealthy countries.

## 1 Introduction

The ‘healthy immigrant effect’ (HIE) suggests recent migrants tend to have better health than the natives in the country of destination or those staying in the country of origin although migrants tend to have lower socioeconomic position than the native-born population (Domnich et al., 2012; Ichou & Wallace, 2019). This could either be due to selection of migrants (i.e. those with better health potential more often move), or to an extent due to underestimation of the socioeconomic position of migrants (Ichou, 2014; Ichou & Wallace, 2019).

Migrants may also experience worse health due to racism and discrimination faced in the healthcare system and the society (Bailey et al., 2017; Llácer et al., 2007; Lockwood et al., 2018; Van Dyke et al., 2017; Williams et al., 2019). This ‘weathering hypothesis’, that is, increased vulnerability due to cumulative exposure to stressors across the life course, states that exposure to racial discrimination over the life course can have a detrimental effect on health beyond that explained by socioeconomic disadvantage of racialized groups (Geronimus, 1992; Geronimus et al., 2006). While the hypothesis originally was not developed to study the experience of migrants, many may still experience such discrimination in the country of destination.

Studies focusing on male migrants or combining data for all genders tend to ignore reproductive health. This is a substantial research gap as most migrants are in their prime reproductive age. Reproductive health is not only important for women and pregnant people,^1^ but has wider implications on their children and families. Given the increasing number of migrants in Europe (36.5 million foreign citizens were living in EU-countries in 2020 (Eurostat, 2021)), as well as the resulting increase in the share of births among migrant women, a better understanding of their health is needed. Such research may also be of interest for policy makers aiming to improve the quality of services provided for immigrants.

Finally, there is a lack of studies on migrant health from a gender perspective, especially one taking into account interactions of migrant status with other characteristics, such as ethnicity or socioeconomic status (Llácer et al., 2007). Yet, these interlinked characteristics entail different vulnerabilities depending on how they intersect. There is a need to better understand effect modification by socioeconomic status among women, who tend to be poorer than men and thus more prone to the negative effects of socioeconomic position on health (Llácer et al., 2007). Interactions are of interest also, as the ‘healthy immigrant effect’ tends to be stronger among disadvantaged socioeconomic groups (Vang et al., 2017; Zufferey, 2016).

In this study, we examine how migrant women's reproductive health, as measured by perinatal outcomes, compares to that of women born in Finland; whether it depends on the characteristics of the migrant; the country of origin; or the length of time spent in Finland. Thus, unlike most previous studies, we take into account the country of origin (income level of the country, and the specific country of origin) and whether it interacts with the women's current socioeconomic status when predicting reproductive health. This analysis helps us understand whether one's socioeconomic situation may moderate the effect of migration status. Finally, HIE predicts recent migrants manifest the largest health advantages, and the weathering hypothesis also suggests worsening health across time due to accumulation of risk exposures. Therefore, we test whether the length of time spent in Finland is associated with perinatal outcomes. Our results are more nuanced than in most previous literature, which often treats all migrants as one group, does not interact migrant status with socioeconomic status, and cannot follow individuals over time. Importantly, we are also able to adjust our analyses for several factors such as women's age, relationship status, parity, body mass index (BMI), and smoking during pregnancy that may confound the associations.

The study examines migrant reproductive health also from a theoretical perspective. We combine the HIE framework with the weathering hypothesis to better understand why some migrant groups fare better and others worse than the native-born population. The results of this study are thus of interest beyond the case study context of Finland, as they provide new evidence on migrant health differentials from a wider theoretical perspective than before. Finland was chosen here due to its exceptional data quality allowing for a longitudinal analysis of the entire population and its interesting welfare state context as described in more detail below.

### 1.1 Theoretical framework: ‘healthy immigrant effect’ and the weathering hypothesis

Research from the HIE perspective rarely focuses on women's or reproductive health (Llácer et al., 2007), even though limited evidence suggests women seem to enjoy less of a positive effect than men (Ichou & Wallace, 2019; Zufferey, 2016). Often, such studies examine all-cause mortality or chronic illness. Yet, a review in Canada showed HIE was less often observed for reproductive health outcomes (Vang et al., 2017), underlining the importance of widening the perspective to better understand the health challenges of migrant women.

The extent of HIE likely depends on the migrants’ standard of living in the host country, and previous life experiences with some groups experiencing health advantages and others disadvantages (Wallace & Wilson, 2021). Studies treating all migrants as one group may thus overlook important variations by country of origin (Villalonga-Olives et al., 2017). For instance, reproductive health studies show migrants from countries culturally similar to the host country tend to have *better outcomes* than other migrants (Juárez et al., 2017). Those from more different countries may have worse access to antenatal care and experience poorer quality of care (Merry et al., 2013), which could overrun the effects of health selection among migrants.

Discrimination may also explain why some migrants do not show any HIE. Racism has a detrimental impact on general health (Bailey et al., 2017; Paradies et al., 2015; Williams et al., 2019) and reproductive health (e.g. Prather et al., 2016). In the US, black people have worse health than white people beyond what is expected based on socioeconomic differences alone, probably due to accumulated life-time stress from discrimination and racism (Geronimus, 1992; Geronimus et al., 2006). This deterioration of health over time among racialized groups may also apply to many migrants.

In addition to the mechanisms following from HIE and the weathering hypothesis, migrant health may also vary depending on whether the individual is a refugee or a voluntary migrant (Hollander et al., 2011; Porter & Haslam, 2005; Wanigaratne et al., 2016), although others did not find such differences (Juárez et al., 2018). Refugees often experience traumatic events in the country of origin and during the journey to the host country. Furthermore, they may struggle integrating into the job market, for example due to laws preventing them from working while waiting for an asylum decision. Communicating with healthcare professionals may also be difficult due to lack of relevant language skills (Merry et al., 2013), although this could be the case for some voluntary migrants too.

### 1.2 Previous literature on migrant reproductive and perinatal health

In this study, we focus on perinatal outcomes, i.e. preterm birth, unplanned C-section, episiotomy, and spontaneous vaginal birth. Preterm birth is often caused by health problems during pregnancy, such as infections or uteroplacental concerns (Goldenberg et al., 2008). An unplanned (emergency) C-section is associated with an increased risk of subsequent pain, wound infections and post-traumatic stress disorder symptoms compared to elective C-sections (Rowlands & Redshaw, 2012). Many episiotomies are unnecessary, even harmful (Amorim et al., 2017; Serati et al., 2019). Unlike the other outcomes, a spontaneous vaginal birth signifies the individual entered labour and delivered without interventions. Such births are associated with fewer health concerns after birth (Rowlands & Redshaw, 2012). Differences in these measures of reproductive health may also reflect the ease/difficulty of navigating the healthcare system.

*Preterm birth.* The main known risk factors for preterm births include infection; placental, uterine and foetal conditions; micronutrient deficiencies; stress; tobacco exposure; and socio-demographic factors such as older maternal age, disadvantaged socioeconomic position and ethnic minority background (Goldenberg et al., 2008; Vogel et al., 2018). Yet, according to some estimates, causes are unknown for up to 65% of all preterm births (Vogel et al., 2018). Postpartum, preterm births are associated with poorer mental health for mothers (Anderson & Cacola, 2017; McDonald et al., 2014; Shapiro et al., 2013), and poorer health and developmental outcomes for the child (Chehade et al., 2018; Vogel et al., 2018).

In Finland in 2004–14, those from Sub-Saharan Africa, South and East Asia had a higher risk of preterm birth than women of Finnish origin, but no significant differences were found among other world regions (Bastola et al., 2020). In Sweden an increased risk of preterm birth was found among mothers from Eastern and Central Europe, Asia and Africa in 1982–2006 (Li et al., 2013). In contrast, in the UK migrants had a lower risk of preterm birth (Opondo et al., 2020).

Smaller sub-national studies show similar effects. In Ontario, Canada, refugees had a higher risk of preterm birth than voluntary migrants (Wanigaratne et al., 2016). In Washington State, Laotian, Cambodian and Vietnamese women had a higher risk of preterm birth than non-Hispanic white women (Cripe et al., 2011). In the French district of Seine-Saint-Denis, the risk of preterm birth was higher among women from the overseas French districts and sub-Saharan Africa (Zeitlin et al., 2004).

*Caesarean section.* While many previous studies do not distinguish between elective and unplanned (emergency) C-sections, we focus on unplanned procedures, as they tend to be associated with worse health outcomes than elective C-sections (Rowlands & Redshaw, 2012).

A meta-analysis in high-income countries found higher unplanned C-section rates for North African/West Asian and Latin American migrants. Limited evidence was available to explain these patterns, but commonly cited risk factors included language barriers, disadvantaged socioeconomic position, poor maternal health, gestational diabetes, high BMI and inadequate prenatal care (Merry et al., 2013).

In Finland, women from South and East Asia, Sub-Saharan Africa, Middle East and Latin America had a higher risk of unplanned C-section than women of Finnish origin, whereas those of Russian origin had a lower risk (Bastola et al., 2020; Malin & Gissler, 2009). In Sweden, those from culturally similar countries were less likely to have unplanned C-sections than those from culturally different countries. Income and employment status did not explain the differences (Juárez et al., 2017). The risk of unplanned C-sections among migrant women increased with length of stay in Sweden, and women from countries where C-section rates are high were more likely to have the procedure (Juarez et al., 2018). In Sweden and Norway, Somali-born women had an elevated unplanned C-section risk (Råssjö et al., 2013; Vangen et al., 2002).

A sub-national study in Australia showed women from South and Central Asia had a higher unplanned C-section risk than non-indigenous Australian women (Agius et al., 2018). On the contrary, studies in Berlin (David et al., 2015, 2018) and Bielefeld in Germany (Miani et al., 2020), found no differences in unplanned C-section rates of migrants. However, they studied all migrants as one group, which might mask differences.

*Episiotomy.* Episiotomy is an incision made at the perineum during labour to aid delivery by making the vaginal opening larger. Differences in episiotomy rates are of interest, as it has been questioned whether the operation results in any benefit (Amorim et al., 2017), or that beneficial effects occur only under few circumstances (Serati et al., 2019). High rates of episiotomy could thus be an indicator of higher-than-average difficulties or complications during labour, more frequent non-necessary interventions, or both.

Few studies investigate the risk of episiotomy among migrants. The existing studies found an increased risk among some migrant groups in Austria (Oberaigner et al., 2013) and Australia (Dahlen et al., 2013; Hennegan et al., 2014). However, only one of these (Hennegan et al., 2014) adjusted for any socioeconomic variables. Finally, a descriptive study in Australia found a higher episiotomy rate among migrants who had experienced female genital mutilation (FGM) (Davis & Jellins, 2019). This is of interest, as in Finland FGM rates are high (69% according to self-reports) among women born in Somalia (Koukkula et al., 2016).

*Interactions.* While disadvantaged socioeconomic position is associated with a risk of negative reproductive health outcomes, such as preterm birth (e.g. Kramer et al., 2000), few reproductive health studies examine interactions between migrant status and socioeconomic position. In Switzerland, those with high education were less likely to have a planned or unplanned C-section than those with lower education in all migrant groups except African women, for whom the association was the opposite, but these differences were not statistically significant (Merten et al., 2007). Women with high education were less likely to have a preterm birth among all migrant and non-migrant women, but higher education was particularly protective among those from Latin America and Balkan/Turkey regions (Merten et al., 2007).

### 1.3 Finnish migration context

Migration to Finland has increased rapidly since the 1990s. Within our study period, the share of the population born abroad rose from 2.6% (n = 136,203) in 2000 to 6.8% (n = 372,802) in 2017 (Statistics Finland, 2021). The largest migrant groups in 2017 were those born in Russia or the former Soviet Union, Estonia, Sweden, Iraq, Somalia, China and Thailand (Statistics Finland, 2021). Many Somali and Kurdish migrants from Iraq are refugees (Castaneda et al., 2012), whereas some of those born in Sweden are likely to be children born to parents of Finnish background, who have since returned to Finland. The share of children born to women of migrant origin increased from 4.2% in 2000 to 11.9% in 2017 (Statistics Finland, 2022). Thus, we need to understand the health care needs of pregnant immigrants in order to improve their health. More generally, it is of interest to understand whether migrants’ perinatal health is affected in the context of a Nordic welfare state, where all residents have access to publicly funded prenatal care.

### 1.4 Aims and research questions

Migrant reproductive health is a complex phenomenon. The strength and direction of its relationship to that of the native-born population likely depends on the socio-demographic characteristics of the migrant, the host country and the length of time spent there, reasons for migration, and the outcome studied. We expect perinatal outcomes of migrants to vary depending on their country of birth, as any ‘healthy immigrant effect’ may be more likely among those from countries culturally and economically similar to Finland. Following the weathering hypothesis, racialized migrants may experience worse perinatal health due to discrimination, difficulties navigating the healthcare system, and poorer job market experiences. Refugee background may add another layer of cumulative exposure to stress and risk factors, which could be associated with worse perinatal outcomes. These negative effects are likely amplified for those in a disadvantaged socioeconomic position, whereas those with more resources may fare better. These relationships may change over time. HIE tends to attenuate over time, and similarly the weathering hypothesis suggest the negative health effects of discrimination accumulate.

There is an overall lack of research on reproductive health of migrant women using nationally representative longitudinal data. Most prior studies include migrants as one group or in a few large geographic groups, which may mask variation between more specific countries of origin. Moreover, studies interacting migrant origin with individual-level socioeconomic status are rare. Finally, the effect of duration of stay in the host country on migrant reproductive health outcomes is not well understood. We aim to fill these gaps in the literature by answering the following research questions:

• Are there differences in perinatal outcomes between migrants and those born in Finland?
•Do these associations vary by (the income level of) country of birth (COB)?
•Does current household income moderate this association?
•Does the association depend on time lived in Finland?

The paper contributes to the literature in multiple ways. It examines an understudied population and understudied health outcomes using a longitudinal full population database. It improves our understanding of the HIE and weathering hypotheses, testing whether these associations depend on the length of time spent in Finland, and the extent to which individual socioeconomic status modifies the association between COB and perinatal health outcomes.

## 2 Data and methods

### 2.1 Data

We obtained administrative register data for years 2000–17. Data on women's and their partners' socio-demographic characteristics, country of birth and time of migration were obtained from Statistics Finland. The Finnish Institute for Health and Welfare's Medical Birth Register contains all births in Finland with information about previous pregnancy outcomes, current pregnancy and its monitoring, and any complications during delivery (The Finnish National Institute for Health and Welfare, 2019). These data were linked using women's unique personal identification code, which every resident in Finland has. Our analytic sample includes all women aged 15–45 who gave birth at least once during the follow up period and had moved to Finland in the calendar year preceding the birth or earlier (N_women_ = 491,532; N_births_ = 948,616).

Statistics Finland Board of Ethics (permit TK-53-339- 13) approved the use of pseudonymised register data for this research. The data were collected for routine administrative registration purposes and, therefore, informed consent of the participants was not required.

*Outcome variables.* We examine *preterm birth* (before 37 weeks of gestation); and the likelihood of experiencing interventions during labour*: unplanned C-section* (i.e. the decision of C-section was made after the delivery started), and *episiotomy*. Finally, we examine the likelihood of *spontaneous vaginal delivery,* that is, a vaginal delivery that was not induced, nor assisted with breech extraction, forceps or vacuum extraction.

*Main co-variates.* We used two groupings of *country of birth*: country's income level (low-, lower-middle-, upper-middle- or high-income economies based on World Bank's classifications in 2017^2^); and individual countries including the ten largest mothers' COBs (in addition to Finland) in terms of the number of childbirths observed in our analytic sample. These countries include (in the order from the largest to the smallest group) Sweden, Former USSR & Russia,^3^ Estonia, Somalia, Iraq, Former Yugoslavia, Thailand, China, Vietnam, and Turkey (see Appendix Table 1).

*Socioeconomic status* is measured using household income in the year before the delivery (or the previous year if missing (n = 2670)). Based on the Tax Administration's database, household income consists of wages, salaries, entrepreneurial income, pensions, unemployment benefits, and some of the other social security benefits. We first divided income by the weighted sum of household members according to the modified OECD equivalence scale and then calculated annual quintile groups. We chose income rather than occupational social class or education to measure SES, because the latter two indicators are more often missing and unreliable among migrants, whose educational achievements, for instance, may have taken place outside Finland and are thus not included in the registers. In addition, we chose to study income over other measures of social class, as it is measured at the household level, and thus makes variation e.g. due to parental leave periods less dramatic.

Finally, for migrant women, we also examine *time lived in Finland* at the time of each birth. It distinguishes between recent (less than 2 years ago), relatively recent (2–5 years ago), relatively long-term (6–9 years ago) and established long-term (10 or more years) migrants (Vang et al., 2017).

*Control variables.* We control for sociodemographic variables measured in the year of each delivery, including woman's age, number and outcome (live birth, abortion, or miscarriage) of her previous pregnancies, smoking during pregnancy (non-smoker, smoked during pregnancy, or smoking data missing), her body mass index (BMI) at antenatal appointments (<18.5, 18.5–24.9, 25–29.9, 30 or more, or BMI missing), and relationship status (married or cohabiting vs. not in union). A year before childbirth, we measured whether or not the partner of the mother was born in Finland, because children from exogamous marriages in Finland have been shown to suffer from worse health outcomes than those from endogamous marriages (Loi et al., 2019). In addition, we adjust for the year of child's birth, child's sex and in which of the 20 hospital districts of Finland the delivery took place.

Analytic sample selection. Our analytic sample excludes multiple births (n = 28,839), deliveries where the COB of the mother was unknown (n = 972), data on household income was missing (n = 3683), mother's parity (n = 703) or gestational age (n = 2210) was missing, mode of delivery (n = 249), number of previous miscarriages or abortions (n = 1002), sex of the child (n = 31), mother's relationship status (n = 2138), or the hospital district was not known (n = 548).

Overall, the level of missing data was low (at most around 1% of births) and did not vary by migrant status. The only exception was household income, which was missing for around one percent of migrant women compared to 0.3% of those born in Finland; and relationship status, which was missing for around one percent of migrants compared to 0.2% among those born in Finland. Comparing included and excluded cases, there were few differences in the proportion experiencing an unplanned C-section or episiotomy, but some differences were found for the other two outcomes: 7.5% of excluded births were preterm compared to 4.5% of included cases; and 67.8% of excluded births were spontaneous vaginal births compared to 75.5% of included births. However, given the small absolute numbers of excluded cases (n = 10,565) in relation to retained ones (n = 984,616), we do not expect this to impact the conclusions drawn from our analyses.

### 2.2 Methods

In addition to descriptive statistics, we conducted multilevel binary logistic regression models including a woman-level random intercept accounting for any time-invariant woman-level propensity affecting each perinatal outcome, as each woman may have more than one birth (equation (1)).

(Eq. 1) $${logit(\mathrm{Pr}(Y}_{ij}=1))=\alpha_{0}+a_{0i}+\beta_{1}x_{ij}+\beta_{2}x_{i}$$

where the mother-specific random intercept *a*_*01*_ accounts for mother-level time-invariant unobserved characteristics and is assumed to be normally distributed. Vector of time-variant characteristics is represented by ***x***_*ij*_ and that of time invariant characteristics by ***x***_***i***_. The odds estimated in our models are subject-specific meaning they will depend on the latent propensity of the mothers to experience each outcome. Thus, characteristics that vary between births (e.g., birth year), represent mother-adjusted associations between births. Time-invariant characteristics (e.g., mother's country of birth), represent differences between similar mothers (in terms of observed and unobserved characteristics), who differ only on the variable of interest (Austin & Merlo, 2017; Rabe-Hesketh & Skrondal, 2008).

### 2.3 Analytic strategy

We conducted separate models by the two groupings of COB: the income level of the country of origin, and the ten largest COBs as separate countries. We tested interactions between the income level of the COB and household income quintile. Finally, we ran multilevel binary logistic regression models with a woman-level random intercept including an interaction between the number of years each migrant had spent in Finland and COB.

All regression models controlled for mother's age, sex of child, the number of previous abortions, miscarriages, and live births (i.e., parity), mother's BMI and smoking during pregnancy, relationship status, partner being born in Finland/abroad, child's year of birth and hospital district.

During the exploratory analysis stage, we examined different ways of measuring the COB. First, we used UN world regions, which did not add much to our results by COB income level, as poorer regions tended to display similar results to low- and middle-income countries and richer regions to high-income countries. Second, we tried including a wider range of countries than the 10 largest, but sample sizes in each individual country were too small (Appendix Table 1). We also explored different ways of measuring time spent in Finland, but using a continuous variable made it difficult to compare migrants to those born in Finland. Finally, we initially included a fifth outcome: very preterm births (before 28 weeks of pregnancy), but the results were very similar to preterm births (available on request).

## 3 Results

### 3.1 Descriptive statistics

Most births among migrant women were among those from upper-middle-income countries (n = 33,538), and high-income countries (n = 29,605), followed by births among women from lower-middle-income countries (n = 10,046), and low-income countries (n = 9724) (Table 1). The distribution of women and births by country is given in Appendix Table 1 and the distribution of the control variables by outcome in Appendix Table 2.

**Table 1:** The frequency of births by key explanatory variables and their distribution by each perinatal outcome in 2000–2017.

	Preterm birth	Unplanned C-section	Episiotomy	Spontaneous vaginal birth	Total N (births)
*COUNTRY OF BIRTH (COB)*					
*Finland*	4.5	9.2	23.3	75.6	901,703
*COB BY INCOME GROUP*					
*Low-income*	4.7	12.7	19.1	73.6	9724
*Lower-middle-income*	5.5	14.7	21.4	68.2	10,046
*Upper-middle-income*	4.5	9.3	21.5	75.1	33,538
*High-income*	4.4	8.9	21.5	76.1	29,605
*SPECIFIC COB*					
*Sweden*	4.7	9.2	21.7	75.6	14,043
*Former USSR & Russia*	4.3	8.1	21.4	75.4	13,803
*Estonia*	4.2	8.2	19.2	77.8	6978
*Somalia*	4.2	11.9	18.7	75.9	5996
*Iraq*	5.1	9.4	17.3	74.9	3203
*Former Yugoslavia*	3.8	6.3	19.3	80.6	3034
*Thailand*	5.1	12.6	20.1	69.8	2817
*China*	4.7	10.8	31.7	69.3	2129
*Vietnam*	5.3	9.1	23.9	76.1	2045
*Turkey*	4.4	9.4	19.9	76.1	1708
*HOUSEHOLD INCOME QUINTILES*					
*Poorest*	4.9	7.8	19.1	80.3	170,154
*Poor*	4.5	7.4	21.4	80.3	172,677
*Middle*	4.2	8.3	21.5	77.8	182,910
*Richer*	4.3	10.0	21.5	73.9	219,967
*Richest*	4.7	12.0	23.3	68.4	238,908
*TIME LIVED IN FINLAND*					
*Born in Finland*	4.5	9.2	23.3	75.6	901,703
*Moved to Finland <2 years ago*	4.5	12.7	29.7	70.3	9385
*Moved to Finland 2–5 years ago*	4.4	10.9	22.3	73.4	25,837
*Moved to Finland 6–9 years ago*	4.6	9.6	18.1	75.6	15,229
Moved to Finland 10+ years ago	4.8	9.2	19.4	75.8	32,462
**Total N (births)**	**44,558**	**91,765**	**227,260**	**743,362**	**984,616**

Women from low-income and lower-middle-income countries were more likely to experience a preterm birth or an unplanned C-section, and less likely to experience a spontaneous vaginal birth than other women (Table 1). Their episiotomy rates, however, were lower. Women from upper-middle and high-income countries fared similarly to or better than native-born women.

Among individual countries of birth, preterm births ranged from 3.8% among mothers born in the Former Yugoslavia to 5.3% among those born in Vietnam. More than one in ten births ended in an unplanned C-section among women from Thailand (12.6%), Somalia (11.9%) and China (10.8%), compared to around 6%–9% among others. Episiotomy was the least common among those born in Iraq (17.3%), and the most common among those born in Finland (23.3%), Vietnam (24%), and China (32%). Around 70% of births to women born in China and Thailand were spontaneous vaginal deliveries compared to 76% among women born in Finland and around 80% among those born in Estonia and Former Yugoslavia (Table 1).

Recent migrants tended to have worse outcomes than others, except for preterm births (Table 1). Differences by current socioeconomic status, measured by household income in the year before delivery, were inconsistent, with a U-shaped income association in preterm births, and higher rates of unplanned C-section and episiotomy among women with higher income (Table 1).

### 3.2 Preterm births

In models adjusted for various maternal characteristics, women from lower-middle-income countries were more likely to have a preterm birth than those born in Finland (aOR = 1.39, [95%CI = 1.23–1.56]), whereas the other groups showed no differences. In contrast to the descriptive statistics, higher household income was associated with lower likelihood of preterm births (Table 2, full results in Appendix Table 3). In separate analyses for the ten largest COBs, we found few significant differences between women born in Finland and those born in the other countries in their risk of preterm births: only Iraq and Vietnam had significantly elevated odds ratios compared to those born in Finland (aOR = 1.24 [95%CI = 1.00–1.55] and aOR = 1.30 [95%CI = 1.00–1.68], respectively) (Table 3, full results in Appendix table 4). The standard deviation of the random intercept in these two models was 1.72. Exponentiating this value gives the odds of premature birth for someone whose unobserved characteristics are one standard deviation above the mean: 5.58 times of those of an average woman. The intraclass correlation (ICC) of 0.47 indicates that 47% of the variation in the outcome was due to unobserved differences between the mothers (Table 2, Table 3). As the random part of the model is not of main interest in this paper, the interpretation is not repeated for each outcome, but it follows the same logic.

**Table 2:** Likelihood of preterm birth, unplanned C-section, episiotomy or spontaneous vaginal birth by income level of COB and household income, adjusted odds ratios (SEs).

	Preterm birth	Unplanned C-section	Episiotomy	Spontaneous vaginal birth
*COUNTRY OF BIRTH’S INCOME LEVEL*				
*Finland (i.e. non-migrant) (ref.)*	1.00	1.00	1.00	1.00
*Low-income*	1.14 (0.08)	3.22 (0.17)***	2.12 (0.07)***	0.33 (0.02)***
*Lower-middle-income*	1.39 (0.08)***	2.51 (0.12)***	1.16 (0.03)***	0.40 (0.02)***
*Upper-middle-income*	1.00 (0.04)	1.14 (0.03)***	1.07 (0.02)***	0.83 (0.02)***
*High-income*	0.95 (0.04)	0.95 (0.03)	0.99 (0.02)	1.05 (0.03)
*HOUSEHOLD INCOME QUINTILES*				
*Poorest (ref.)*	1.00	1.00	1.00	1.00
*Poor*	0.98 (0.02)	1.00 (0.02)	1.01 (0.01)	0.96 (0.01)**
*Middle*	0.91 (0.02)***	0.99 (0.02)	1.06 (0.01)***	0.94 (0.01)***
*Richer*	0.91 (0.02)***	1.00 (0.02)	1.11 (0.01)***	0.91 (0.01)***
*Richest*	0.92 (0.02)**	0.95 (0.02)*	1.12 (0.01)***	0.91 (0.02)***
*Standard deviation of random intercept a*_*0i*_	1.72	1.85	0.52	2.50
*ICC*	0.47	0.51	0.08	0.66

**Notes:** Controlling for mother's age at birth, parity, child's sex, mother's BMI and smoking during pregnancy, previous abortions and miscarriages, partner born abroad, relationship status at birth, hospital district and year of child's birth (full results in Appendix Table 3). *p < 0.05; **p < 0.01; ***p < 0.001.

**Table 3:** Likelihood of preterm birth, unplanned C-section, episiotomy or spontaneous vaginal birth by COB for the 10 largest origin countries and women born in Finland, adjusted odds ratios (SEs).

	Preterm birth	Unplanned C-section	Episiotomy	Spontaneous vaginal birth
*COUNTRY OF BIRTH*				
*Finland (ref.)*	1.00	1.00	1.00	1.00
*Sweden*	1.03 (0.06)	1.06 (0.05)	0.99 (0.02)	0.89 (0.04)**
*Former USSR and Russia*	0.94 (0.05)	0.86 (0.04)**	0.98 (0.02)	1.21 (0.05)***
*Estonia*	0.94 (0.07)	0.93 (0.06)	1.01 (0.04)	1.14 (0.07)*
*Somalia*	0.96 (0.09)	3.49 (0.45)***	3.24 (0.14)***	0.32 (0.02)***
*Iraq*	1.24 (0.14)*	1.67 (0.16)***	1.33 (0.08)***	0.47 (0.04)***
*Former Yugoslavia*	0.85 (0.11)	0.90 (0.10)	1.27 (0.07)***	0.97 (0.09)
*Thailand*	1.21 (0.13)	1.92 (0.16)**	1.01 (0.06)	0.51 (0.04)***
*China*	1.03 (0.13)	1.08 (0.11)	1.42 (0.08)***	0.79 (0.08)*
*Vietnam*	1.30 (0.17)*	1.36 (0.16)**	1.38 (0.09)***	0.76 (0.08)*
*Turkey*	1.07 (0.16)	1.36 (0.17)*	1.18 (0.09)*	0.65 (0.08)***
*Standard deviation of random intercept a*_*0i*_	1.72	1.85	0.52	2.49
*ICC*	0.47	0.51	0.08	0.65

**Notes:** Controlling for household income, mother's age at birth, parity, child's sex, mother's BMI and smoking during pregnancy, previous abortions and miscarriages, partner born abroad, relationship status at birth, hospital district and year of child's birth (full results not shown due to control variable results being very similar to those shown in Appendix Table 4). *p < 0.05; **p < 0.01; ***p < 0.001.

### 3.3 Unplanned C-section

Women born in low-income or middle-income countries were more likely than the native-born to experience an unplanned C-section (aORs 3.22 [95%CI = 2.91–3.57], 2.51 [95%CI = 2.29–2.75] and 1.14 [95%CI = 1.07–1.21] for low, lower-middle and upper-middle-income respectively, Table 2 & Appendix Table 3).

When individual COBs were examined, women from Iraq (aOR = 1.67 [95%CI = 1.39–2.02]), Somalia (aOR = 3.49 [95%CI = 3.05–4.00]), Vietnam (aOR = 1.36 [95%CI = 1.09–1.71]), Thailand (aOR = 1.92 [95%CI = 1.63–2.28]), and Turkey (aOR = 1.36 [95%CI = 1.07–1.75]) were more likely than women born in Finland to experience an unplanned C-section. The other countries did not differ from Finland apart from the Former USSR, where the risk was lower (aOR = 0.86 [95%CI = 0.78–0.94]) (Table 3 & Appendix Table 4).

### 3.4 Episiotomy

While women from low-income countries had fewer episiotomies in the descriptive statistics (Table 1), the adjusted association reversed. They were twice more likely than women from Finland to have an episiotomy. Those from lower-middle- and upper-middle-income countries also had increased odds (aOR = 1.16 [95%CI = 1.09–1.23] and aOR = 1.07 [95%CI = 1.04–1.11], respectively). Interestingly, higher household income was associated with elevated odds of episiotomy (Table 2 & Appendix Table 3).

When individual COBs were analysed, women born in Somalia had the most elevated odds of episiotomy (aOR = 3.24 [95%CI = 2.97–3.53]), followed by women from China (aOR = 1.42 [95%CI = 1.28–1.58]), Vietnam (aOR = 1.38 [95%CI = 1.22–1.56]), Iraq (aOR = 1.33 [95%CI = 1.19–1.49]), and the Former Yugoslavia (aOR = 1.27 [95%CI = 1.14–1.42]) (Table 3 & Appendix Table 4).

### 3.5 Spontaneous vaginal births

The likelihood of spontaneous vaginal birth was the lowest among migrants from low-income countries and the highest among those from high-income countries and Finland. In contrast, higher household income was associated with reduced odds of spontaneous vaginal delivery (Table 2). Among individual COBs, Estonia (aOR = 1.14 [95%CI = 1.01–1.27]) and Former USSR & Russia (aOR = 1.21 [95%CI = 1.12–1.32]) had higher odds than Finland. Women from Somalia (aOR = 0.32 [95%CI = 0.28–0.37]), Iraq (aOR = 0.47 [95%CI = 0.39–0.56]) and Thailand (aOR = 0.51 [95%CI = 0.43–0.60]) were the least likely to experience a spontaneous vaginal birth (Table 3 & Appendix Table 4).

### 3.6 Household income and country of birth interactions

**Fig. 1:**
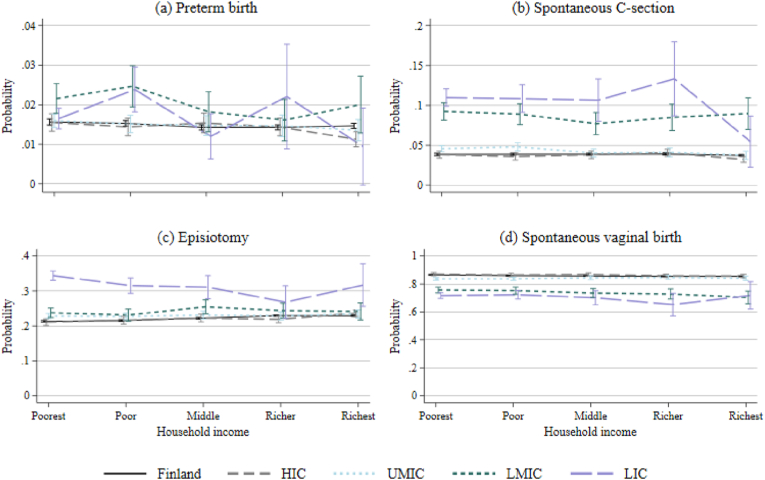
Predicted probabilities and 95% confidence intervals of (a) preterm birth, (b) unplanned C-section, (c) episiotomy and (d) spontaneous vaginal delivery, interaction between COB income level and household income. **Notes:** Y-axis varies. Controlling for mother's age, sex of child, previous abortions and miscarriages, parity, mother's BMI, smoking during pregnancy, relationship status and partner being born in Finland/abroad, child's year of birth and hospital district. HIC = high income, UMIC = upper middle-income, LMIC = lower middle-income and LIC = low income. P-values from joint Wald-tests associated with interactions by panel: (a) p = 0.041; (b) p = 0.045; (c) p = 0.003; and (d) p = 0.472).

There was an interaction (p = 0.041) between COB income level and one's household income quintile in the preterm birth model. Fig. 1(a) shows predicted probabilities of this interaction, full results are in Appendix Table 5. The probabilities of a preterm birth among women born in Finland, and migrants from high-income or upper-middle-income countries were mostly similar across income groups. The probabilities fluctuated more for the low-income and lower-middle-income country groups by household income, but no clear pattern of increase or decrease emerged.

While women from low-income countries generally had the highest probability of an unplanned C-section, the probability was reduced close to the levels of native Finns and other richer countries if the women belonged to the richest household income quintile (Fig. 1(b)). However, there were only 159 women in this group.

The probability of episiotomy was similar among women from Finland, high-income and upper-middle-income countries across household income (Fig. 1(c)). The highest risk was observed among those from low-income countries, particularly if they also had low household income.

There was no interaction between household income and COB for spontaneous vaginal birth (Fig. 1(d)).

### 3.7 Time since migration

**Fig. 2:**
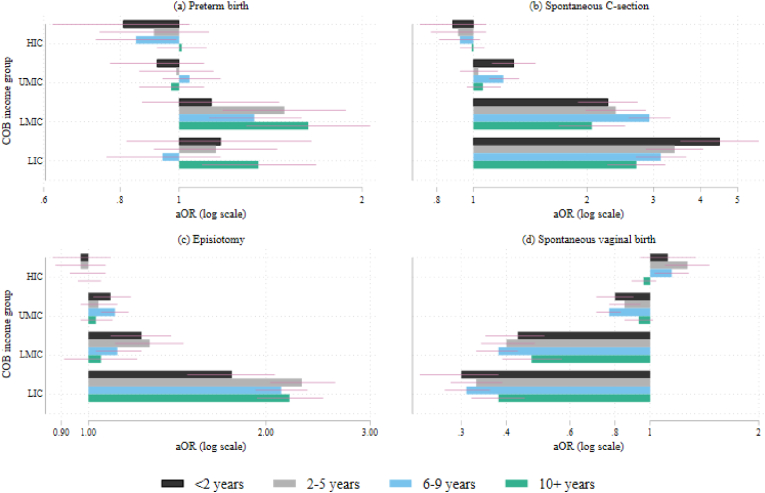
Likelihood of preterm birth, unplanned C-section, episiotomy or spontaneous vaginal birth by time lived in Finland, adjusted odds ratios on log scale and 95% confidence intervals, reference group: born in Finland. **Notes:** X-axis varies. Controlling for mother's age, household income, sex of child, previous abortions and miscarriages, parity, mother's BMI, smoking during pregnancy, relationship status and partner being born in Finland/abroad, child's year of birth and hospital district; HIC = high income, UMIC = upper middle-income, LMIC = lower middle-income and LIC = low income. *p < 0.05; **p < 0.01; ***p < 0.001; joint Wald-test for the interaction in each model p < 0.001.

The odds from multilevel logistic regression models focusing on the time spent in Finland are shown in Fig. 2 (full results in Appendix Table 6). For the most part, there were no differences between women born in Finland, high-income countries, and upper-middle-income countries. For spontaneous C-section, the higher risk among migrants from low-income and lower-middle-income countries attenuated, but stayed high over time. For the other outcomes, the risk either slightly increased (preterm birth, episiotomy among women from low-income countries) or fluctuated (spontaneous vaginal birth, episiotomy among women from lower-middle-income countries).

## 4 Discussion

### 4.1 Migrant reproductive health varies by country of birth

We compared perinatal outcomes of women who had migrated to Finland to those born in Finland using total population data of around half-a-million women and almost a million births in 2000–2017. Overall, we found only a very modest healthy immigrant effect for a few countries. Most perinatal outcomes were worse for migrants from lower income countries, whereas women from other high-income countries fared similarly to native-born women. This result is potentially in line with the weathering hypothesis (Geronimus, 1992; Geronimus et al., 2006), although we could not formally test this due to lack of data e.g. on experiences of discrimination. These results show the immigrant health advantage may not hold for reproductive health, potentially due to difficulties with discrimination and navigating the health system, but more research is needed in other contexts and with different sets of explanatory variables to confirm this.

Examining individual COBs gives a detailed picture of these complex associations. Some countries displayed a modest healthy immigrant effect. Those born in Estonia, Sweden, and the Former USSR & Russia fared either better than or similarly to women born in Finland in models adjusting for confounders. This advantage compared to other migrants may be due to positive health selection of migrants and because these wealthy neighbouring European countries are culturally relatively similar to Finland (cf. Juárez et al., 2017). In particular, Swedish is an official language in Finland, and Estonian is linguistically close to Finnish. Thus, women from these countries may struggle less with language barriers and therefore have fewer difficulties navigating the prenatal healthcare system than those born elsewhere (Merry et al., 2013).

In line with previous studies that indicate migrant women born in Africa (Bastola et al., 2020; Li et al., 2013; Råssjö et al., 2013; Vangen et al., 2002; Zeitlin et al., 2004) and parts of Asia (Agius et al., 2018; Bastola et al., 2020; Cripe et al., 2011; Li et al., 2013) might have a higher risk of negative reproductive health outcomes, those from Iraq and Vietnam fared worse than women born in Finland in all outcomes, and those from Somalia and Turkey fared worse in three: unplanned C-section, episiotomy and spontaneous vaginal birth. Those born in Somalia had the most extreme risks. Women from China fared worse in episiotomy and spontaneous vaginal birth, and those from Thailand in unplanned C-sections and spontaneous vaginal birth.

These results support the weathering hypothesis, as ethnic minorities and those from culturally and linguistically different countries might be more often subjected to racism and discrimination, which can have a detrimental impact on (reproductive) health (Bailey et al., 2017; Geronimus, 1992; Geronimus et al., 2006; Paradies et al., 2015; Prather et al., 2016; Williams et al., 2019). In addition, women from Somalia, Iraq and Kurdish women from Turkey are likely to be refugees, which has been linked with poorer reproductive health outcomes, possibly because of accumulation of trauma and discrimination, and disadvantages in the labour market (Hollander et al., 2011; Porter & Haslam, 2005; Wanigaratne et al., 2016). Finally, the relatively high rate of FGM among women of Somalian origin living in Finland (Koukkula et al., 2016) may partly explain their increased risk of episiotomy and C-section (Davis & Jellins, 2019).

### 4.2 Individual socioeconomic position and migrant perinatal health

One's own socioeconomic position modified the relationship between COB and perinatal outcomes less than we expected. For instance, the risk of adverse outcomes among women from low- and lower-middle-income countries was not typically offset by an advantaged individual-level socioeconomic status. We only observed such offset of risk for unplanned C-sections and to a lesser extent episiotomy, where high household income meant that the risk of these outcomes among migrants from poorer countries approached that of those born in Finland. The lack of associations may in part be due to the low number of migrants from low-income countries in the highest categories of household income: only 159 women from a low income country lived in a household that belonged to the top 20% of earners. It could also be due to factors to do with selection of migrants according to the country of origin and reason for migration. In addition, in a welfare state higher income does not necessarily mean better access to or quality of healthcare received. It may be that other processes, such as trauma, discrimination and racism are more important. Future research should determine, whether measuring other dimensions of socioeconomic position, such as education, yields different results.

### 4.3 No clear effects over time lived in Finland

The HIE advantage based on health selection of immigrants may wear off over time (Domnich et al., 2012; Ichou & Wallace, 2019), although some studies found no clear effects (Juárez & Hjern, 2017). Following the weathering hypothesis (Geronimus, 1992; Geronimus et al., 2006), the health outcomes for those experiencing frequent discrimination deteriorate over time as the effects accumulate. While we only found a modest HIE for a few countries and thus could not assess potential attenuation of the association over time, we found some modest evidence for the weathering hypothesis, as the risk of preterm birth was higher among women from low- and lower-middle-income countries the longer they had lived in Finland. Yet, this result could be also partly driven by changes in the composition of migrant populations moving to Finland over time as a function of, for instance, macroeconomic conditions and locations of conflicts at any given time.

The reason preterm birth was the only outcome showing this pattern is perhaps that it is more directly related to mothers' and foetal health than the other outcomes, which are interventions during labour and may be linked to the ability to use the healthcare system and communicate with healthcare professionals. While Finland's universal low-cost healthcare system may make it somewhat easier for migrants to use the system compared to countries like the US, where no such system exists, many migrants nevertheless use the system less than those born in Finland (Kemppainen et al., 2018; Rask et al., 2016). The ability to use the system and communicate with the professionals likely increases over time spent in the host country, which may explain why increasing disadvantage over time in Finland were not found for these outcomes.

### 4.4 Strengths and limitations of the study

The strengths of the study include the use of a full administrative population database and long follow up period of 18 years with all participants observed throughout for as long as they live in Finland. The advantages of using administrative data to study migrants are clear, as migrants are often hard to reach in surveys. The use of administrative data ensures an adequate number of cases for most statistical analyses even if separated into individual countries of origin. This is important, because the selection of migrants is likely different depending on the country of origin, and the mechanism may be linked with health outcomes (Ichou, 2014; Ichou & Wallace, 2019). These data also allow for controlling for an extensive range of socio-demographic factors longitudinally. Finally, while many migrant health outcomes may be difficult to measure using healthcare data due to lower use of services (Kemppainen et al., 2018; Rask et al., 2016), virtually all births take place in a healthcare setting in Finland (Zeitlin et al., 2010). Thus, register data for pregnancy and birth complications is likely more reliable than other migrant healthcare data.

There were limitations in our study. While we chose household income as the socioeconomic measure least likely to be biased for migrants in registers, it may be that we underestimate some migrants' socioeconomic status if they struggle integrating into the Finnish labour market (Aalto et al., 2014; Fornaro, 2018) and thus have jobs with low pay given their previous experience or level of education. In addition, income alone is unable to measure socioeconomic position as a multifaceted phenomenon. Moreover, we classified so called second generation migrants as native born. However, as they only make-up about 1.3% of the Finnish population (Official Statistics of Finland, 2019), it is unlikely to bias our results. Furthermore, while we can make an educated guess as to the reason for migration (refugee vs. voluntary) based on the COB, it was not measured directly. Moreover, a more complete picture of reproductive health would require also using outcomes not directly related to pregnancy and labour such as sexually transmitted infections, contraceptive use and pregnancies not ending in live births. However, as discussed above, this could have introduced bias, as migrants may be less likely to use healthcare services other than those related to giving birth. Finally, the exact manifestation of the ‘healthy immigrant effect’ may differ by the host country (Villalonga-Olives et al., 2017). The potential mechanisms linking the institutional context to migrant reproductive health should be examined in cross-country comparative studies. Future studies should also investigate changes in migrant health over calendar time and how these health changes relate to policies among different groups of migrants. Overall, we believe the strengths of the study outweigh any possible limitations, and that it provides robust evidence on migrant reproductive health, relevant beyond the Finnish context.

## 5 Conclusions

Migrant reproductive health is a complex phenomenon depending on one's COB, selectivity of the migratory flows and circumstances in the host country. We show that for perinatal outcomes HIE hardly exists, apart for migrants from a few high-income countries. On the contrary, women from poor countries tended to fare worse than native-born women. Discrimination, language barriers in navigating healthcare systems or refugee experiences are among the possible mechanisms explaining the worse outcomes of migrants from poorer countries. One's own socioeconomic position in many cases did not modify the association between COB and perinatal outcomes, and the effect of time spent in Finland was also modest. The inequalities observed in a global scale in countries' economic outcomes tend to reproduce themselves as health inequalities in more local level among migrants. These results can help policymakers to provide better care for the diverse migrant populations.

## Author contributions

**Heini Väisänen:** Conceptualization; Data curation; Formal analysis; Methodology; Project administration; Writing - original draft; Writing - review & editing.

**Hanna Remes:** Conceptualization; Methodology; Writing - original draft; Writing - review & editing.

**Pekka Martikainen:** Conceptualization; Methodology; Resources; Writing - review & editing.

## Ethics

This study is based on secondary data collected for administrative and statistical purposes. We have obtained permission to access these data from Statistics Finland (TK-53–339-13) and Findata Health and Social Data Permit Authority (THL/2180/14.02.00/2020) after consideration by the ethical boards of these statistical authorities. The study complies with the national legal framework for accessing anonymous personal data for scientific research carried out in public interest. The legal basis is stated in the Finnish Personal Data Act (523/1999), Act on Secondary use of Social and Healthcare data (552/2019), Finnish Statistics Act (280/2004) and the EU General Data Protection Regulation (GDPR). The GDPR permits processing this type of data for research without using the GDPR consent (Art. 9 of the GDPR).

## Declaration of competing interest

None to declare.

## Data Availability

The authors do not have permission to share data.

## Footnotes

1 In the rest of this article we refer to the group we study as ‘women’, because we selected our analytic sample in part based on their gender being recorded as ‘woman’ in the population register.

2 See: https://datahelpdesk.worldbank.org/knowledgebase/articles/906519-world-bank-country-and-lending-groups.

3 It is often not possible to differentiate between the current countries of the Former USSR in the registers. When this was the case, these were combined with Russia, which the largest country in this area. The same applies to those born in the former Yugoslavia. We use the word ‘country’ for these regions, as historically they were single countries.
