# Supplementary material for: Perinatal health among migrant women: A longitudinal register study in Finland 2000-17
Source: SSM Popul Health. 2022 Nov 24;20:101298. doi: 10.1016/j.ssmph.2022.101298 (PMC9712663; doi:10.1016/j.ssmph.2022.101298)
Supplement: Multimedia component 1 [file mmc1.docx]

Appendix table 1. Distribution of births and women by country of birth.

|  | **Births** | | **Women** | |
| --- | --- | --- | --- | --- |
| **COUNTRY OF BIRTH** | *% of all births* | *N* | *% of all women* | *N* |
| Finland | 91.39 | 901,703 | 90.60 | 491,532 |
| LOW-INCOME |  |  |  |  |
| Somalia | 0.61 | 5,996 | 0.44 | 2,406 |
| Afganistan | 0.13 | 1,283 | 0.14 | 752 |
| DRC | 0.07 | 663 | 0.07 | 369 |
| Ethiopia | 0.06 | 564 | 0.06 | 336 |
| Nepal | 0.03 | 279 | 0.04 | 235 |
| Syria | 0.03 | 338 | 0.04 | 227 |
| Tanzania | 0.02 | 171 | 0.02 | 96 |
| Gambia | 0.01 | 111 | 0.01 | 62 |
| Others | 0.03 | 319 | 0.04 | 195 |
| Total (low income) | 0.99 | 9,724 | 0.86 | 4,678 |
| LOWER-MIDDLE-INCOME |  |  |  |  |
| Vietnam | 0.21 | 2,045 | 0.23 | 1,234 |
| India | 0.11 | 1,112 | 0.16 | 845 |
| The Philipines | 0.10 | 991 | 0.13 | 732 |
| Morocco | 0.08 | 813 | 0.08 | 436 |
| Bangladesh | 0.06 | 616 | 0.08 | 411 |
| Pakistan | 0.06 | 550 | 0.06 | 350 |
| Nigeria | 0.05 | 498 | 0.06 | 332 |
| Kenya | 0.05 | 454 | 0.05 | 284 |
| Ukraine | 0.04 | 349 | 0.05 | 266 |
| Ghana | 0.04 | 382 | 0.04 | 227 |
| Sudan | 0.05 | 489 | 0.04 | 213 |
| Myanmar | 0.03 | 290 | 0.04 | 194 |
| Cameroon | 0.02 | 238 | 0.03 | 152 |
| Indonesia | 0.02 | 180 | 0.02 | 121 |
| Angola | 0.02 | 160 | 0.02 | 90 |
| Tunis | 0.02 | 191 | 0.02 | 93 |
| Egypt | 0.01 | 139 | 0.01 | 77 |
| Cambodia | 0.01 | 101 | 0.01 | 64 |
| Others | 0.05 | 448 | 0.05 | 290 |
| Total (low middle income) | 1.02 | 10,046 | 1.18 | 6,411 |
| UPPER-MIDDLE-INCOME |  |  |  |  |
| Former USSR | 1.40 | 13,803 | 1.72 | 9,354 |
| Iraq | 0.32 | 3,203 | 0.31 | 1,694 |
| Former Yugoslavia | 0.31 | 3,034 | 0.30 | 1,656 |
| Thailand | 0.29 | 2,817 | 0.38 | 2,061 |
| China | 0.22 | 2,129 | 0.28 | 1,529 |
| Turkey | 0.17 | 1,708 | 0.18 | 997 |
| Russia | 0.16 | 1,537 | 0.20 | 1,110 |
| Iran | 0.11 | 1,090 | 0.14 | 745 |
| Romania | 0.06 | 557 | 0.07 | 394 |
| Brazil | 0.04 | 410 | 0.05 | 294 |
| Former Serbia & Montenegro | 0.04 | 410 | 0.04 | 197 |
| Sri Lanka | 0.03 | 318 | 0.04 | 194 |
| Appendix table 1. (cont.) | | | | |
|  | **Births** | **Women** |  |  |
| **COUNTRY OF BIRTH** | *% of all births* | *N* | *% of all women* | *N* |
| UPPER-MIDDLE-INCOME (cont.) | |  |  |  |
| Bulgaria | 0.03 | 290 | 0.04 | 216 |
| Algeria | 0.03 | 286 | 0.03 | 141 |
| Bosnia & Herzegovina | 0.02 | 202 | 0.02 | 132 |
| Mexico | 0.02 | 161 | 0.02 | 109 |
| Lebanon | 0.02 | 157 | 0.01 | 76 |
| Peru | 0.01 | 147 | 0.02 | 103 |
| Malesia | 0.01 | 99 | 0.01 | 68 |
| Cuba | 0.01 | 76 | 0.01 | 60 |
| Albania | 0.01 | 74 | 0.01 | 54 |
| Argentina | 0.01 | 74 | 0.01 | 51 |
| Belarus | 0.01 | 69 | 0.01 | 52 |
| Others | 0.09 | 887 | 0.10 | 556 |
| Total (upper middle income) | 3.40 | 33,538 | 4.02 | 21,843 |
| HIGH-INCOME |  |  |  |  |
| Sweden | 1.42 | 14,043 | 1.38 | 7,503 |
| Estonia | 0.71 | 6,978 | 0.93 | 5,030 |
| Germany | 0.11 | 1,117 | 0.13 | 705 |
| Poland | 0.08 | 768 | 0.10 | 545 |
| USA | 0.08 | 771 | 0.08 | 450 |
| Japan | 0.05 | 469 | 0.06 | 322 |
| Hungary | 0.05 | 483 | 0.06 | 315 |
| United Kingdom | 0.05 | 466 | 0.06 | 299 |
| Latvia | 0.04 | 418 | 0.05 | 285 |
| France | 0.04 | 385 | 0.05 | 255 |
| Spain | 0.04 | 403 | 0.05 | 257 |
| Canada | 0.04 | 385 | 0.04 | 200 |
| Lithuania | 0.03 | 253 | 0.03 | 177 |
| Italy | 0.03 | 267 | 0.03 | 173 |
| Norway | 0.03 | 279 | 0.03 | 151 |
| Australia | 0.02 | 199 | 0.02 | 125 |
| The Netherlands | 0.02 | 159 | 0.02 | 99 |
| Former Czechoslovakia | 0.02 | 158 | 0.02 | 99 |
| Denmark | 0.01 | 145 | 0.02 | 92 |
| Switzerland | 0.01 | 146 | 0.02 | 91 |
| Israel | 0.01 | 136 | 0.02 | 83 |
| South Korea | 0.01 | 101 | 0.01 | 78 |
| Greece | 0.01 | 93 | 0.01 | 73 |
| Austria | 0.01 | 108 | 0.01 | 68 |
| Chile | 0.01 | 89 | 0.01 | 64 |
| Saudi Arabia | 0.01 | 94 | 0.01 | 58 |
| Others | 0.07 | 692 | 0.08 | 455 |
| Total (high income) | 3.00 | 29,605 | 3.32 | 18,052 |
| TOTAL | 99.80 | 984,616 | 99.99 | 542,516 |

Appendix table 2. The percentage of births by control variables and outcome in 2000-2017.

|  | **Preterm birth (n=44,558)** | **Unplanned C-section (n=91,765)** | **Episiotomy (n= 227,260)** | **Spontaneous vaginal birth (n=743,362)** | **Total N (N_(births)_= 984,616)** |
| --- | --- | --- | --- | --- | --- |
| *Mother's age at birth* |  |  |  |  |  |
| Mother aged <20 at birth | 5.5 | 7.7 | 40.0 | 80.1 | 23,163 |
| Mother aged 20-24 at birth | 4.5 | 8.3 | 30.3 | 79.3 | 152,497 |
| Mother aged 25-29 at birth | 4.2 | 8.7 | 25.6 | 77.0 | 307,467 |
| Mother aged 30-34 at birth | 4.3 | 9.5 | 20.3 | 74.8 | 311,110 |
| Mother aged 35+ at birth | 5.3 | 11.1 | 15.8 | 70.7 | 190,379 |
| *Number of previous births* |  |  |  |  |  |
| Parity 0 | 5.4 | 14.4 | 42.1 | 64.5 | 406,451 |
| Parity 1 | 3.6 | 6.7 | 14.1 | 81.7 | 332,435 |
| Parity 2 | 3.9 | 4.5 | 5.0 | 84.2 | 147,546 |
| Parity 3+ | 4.9 | 4.4 | 1.6 | 87.2 | 98,184 |
| *Child sex* |  |  |  |  |  |
| Child sex: boy | 4.9 | 10.1 | 23.9 | 74.1 | 503,668 |
| Child sex: girl | 4.1 | 8.6 | 22.0 | 77.0 | 480,948 |
| *Mother's BMI before pregnancy* |  |  |  |  |  |
| Mother BMI <18.5 | 5.8 | 6.7 | 25.5 | 78.5 | 26,802 |
| Mother BMI 18.5-24.9 | 4.2 | 8.2 | 22.7 | 76.5 | 458,611 |
| Mother BMI 25-29.9 | 4.4 | 10.9 | 18.6 | 73.4 | 163,049 |
| Mother BMI 30+ | 5.3 | 14.1 | 14.8 | 69.3 | 91,344 |
| Mother BMI missing | 4.9 | 8.8 | 29.6 | 77.0 | 244,810 |
| *Smoking during pregnancy* |  |  |  |  |  |
| Mother didn't smoke during pregnancy | 4.3 | 9.2 | 23.1 | 75.6 | 813,775 |
| Mother smoked during pregnancy | 5.3 | 10.1 | 23.8 | 75.3 | 146,464 |
| Mother smoking missing | 7.4 | 9.8 | 19.0 | 74.5 | 24,377 |
| *Previous induced abortions* |  |  |  |  |  |
| Never had an abortion | 4.5 | 9.3 | 23.6 | 75.5 | 860,182 |
| Had one abortion | 4.8 | 9.8 | 20.9 | 75.0 | 98,548 |
| Had 2 or more abortions | 5.5 | 9.3 | 15.9 | 76.3 | 25,886 |
| *Previous miscarriages* |  |  |  |  |  |
| Never had a miscarriage | 4.4 | 9.4 | 24.9 | 75.2 | 774,903 |
| Had one miscarriage | 4.7 | 9.0 | 17.7 | 76.7 | 155,408 |
| Had 2 or more miscarriages | 6.0 | 9.2 | 13.2 | 76.4 | 54,305 |
| *Partner's country of birth (year before childbirth)* | | | | | |
| Partner born in Finland | 4.4 | 9.1 | 22.9 | 75.8 | 794,055 |
| Partner born abroad | 4.3 | 9.2 | 20.5 | 76.2 | 71,182 |
| No partner recorded | 5.6 | 11.0 | 25.9 | 72.8 | 119,379 |
| *Mother's relationship status* |  |  |  |  |  |
| Mother married/cohabiting | 4.4 | 9.3 | 22.7 | 75.6 | 913,431 |
| Mother not cohabiting/married | 5.8 | 10.2 | 28.4 | 74.6 | 71,185 |
| **NB. Child's birth year and hospital district not shown.** | | | | | |

Appendix table 3. Likelihood of preterm birth, unplanned C-section, episiotomy or spontaneous vaginal birth by income level of COB and household income, adjusted odds ratios (95% CIs), full results.

|  | **Preterm birth** | | **Unplanned C-section** | | **Episiotomy** | | **Spontaneous vaginal birth** | |
| --- | --- | --- | --- | --- | --- | --- | --- | --- |
|  | *aOR (95% CI)* | *p-value* | *aOR (95% CI)* | *p-value* | *aOR (95% CI)* | *p-value* | *aOR (95% CI)* | *p-value* |
| COUNTRY OF BIRTH’S INCOME LEVEL |  |  |  |  |  |  |  |  |
| Finland i.e. non-migrant (ref.) | 1.00 |  | 1.00 |  | 1.00 |  | 1.00 |  |
| Low income country | 1.14 (1.00–1.30) | 0.057 | 3.22 (2.91–3.57) | <0.001 | 2.12 (1.99–2.27) | <0.001 | 0.33 (0.30–0.37) | <0.001 |
| Lower middle income | 1.39 (1.23–1.56) | <0.001 | 2.51 (2.29–2.75) | <0.001 | 1.16 (1.09–1.23) | <0.001 | 0.40 (0.37–0.45) | <0.001 |
| Upper middle income | 1.00 (0.93–1.08) | 0.974 | 1.14 (1.07–1.21) | <0.001 | 1.07 (1.04–1.11) | <0.001 | 0.83 (0.78–0.88) | <0.001 |
| High income country | 0.95 (0.88–1.02) | 0.174 | 0.95 (0.90–1.01) | 0.138 | 0.99 (0.96–1.03) | 0.714 | 1.05 (0.99–1.11) | 0.104 |
| HOUSEHOLD INCOME QUINTILES |  |  |  |  |  |  |  |  |
| Poorest (ref.) | 1.00 |  | 1.00 |  | 1.00 |  | 1.00 |  |
| Poor | 0.98 (0.94–1.02) | 0.399 | 1.00 (0.96–1.03) | 0.939 | 1.01 (0.99–1.03) | 0.314 | 0.96 (0.93–0.99) | 0.004 |
| Middle | 0.91 (0.88–0.95) | <0.001 | 0.99 (0.96–1.03) | 0.661 | 1.06 (1.04–1.08) | <0.001 | 0.94 (0.91–0.97) | <0.001 |
| Richer | 0.91 (0.87–0.95) | <0.001 | 1.00 (0.97–1.04) | 0.941 | 1.11 (1.09–1.14) | <0.001 | 0.91 (0.88–0.94) | <0.001 |
| Richest | 0.93 (0.89–0.97) | 0.002 | 0.95 (0.92–0.99) | 0.011 | 1.12 (1.09–1.14) | <0.001 | 0.91 (0.88–0.94) | <0.001 |
| CONTROL VARIABLES |  |  |  |  |  |  |  |  |
| *Mother's age at birth* |  |  |  |  |  |  |  |  |
| Mother aged <20 at birth | 0.89 (0.83–0.96) | 0.003 | 0.44 (0.41–0.47) | <0.001 | 0.98 (0.95–1.02) | 0.304 | 3.25 (3.07–3.45) | <0.001 |
| Mother aged 20-24 at birth | 0.90 (0.86–0.93) | <0.001 | 0.68 (0.66–0.70) | <0.001 | 0.96 (0.94–0.97) | <0.001 | 1.84 (1.79–1.89) | <0.001 |
| Mother aged 25-29 at birth (ref.) | 1.00 |  | 1.00 |  | 1.00 |  | 1.00 |  |
| Mother aged 30-34 at birth | 1.10 (1.07–1.14) | <0.001 | 1.49 (1.45–1.52) | <0.001 | 1.05 (1.03–1.06) | <0.001 | 0.55 (0.54–0.56) | <0.001 |
| Mother aged 35+ at birth | 1.46 (1.41–1.51) | <0.001 | 2.35 (2.28–2.42) | <0.001 | 1.11 (1.09–1.13) | <0.001 | 0.28 (0.27–0.29) | <0.001 |
| *Number of previous births* |  |  |  |  |  |  |  |  |
| Parity 0 (ref.) | 1.00 |  | 1.00 |  | 1.00 |  | 1.00 |  |
| Parity 1 | 0.57 (0.56–0.59) | <0.001 | 0.25 (0.25–0.26) | <0.001 | 0.19 (0.19–0.19) | <0.001 | 7.02 (6.88–7.17) | <0.001 |
| Parity 2 | 0.57 (0.55–0.59) | <0.001 | 0.13 (0.12–0.13) | <0.001 | 0.06 (0.05–0.06) | <0.001 | 11.8 (11.4–12.1) | <0.001 |
| Parity 3+ | 0.66 (0.63–0.69) | <0.001 | 0.11 (0.10–0.11) | <0.001 | 0.02 (0.02–0.02) | <0.001 | 17.8 (17.1–18.6) | <0.001 |
| *Child sex* |  |  |  |  |  |  |  |  |
| Child sex: boy (ref.) | 1.00 |  | 1.00 |  | 1.00 |  | 1.00 |  |
| Child sex: girl | 0.80 (0.78–0.82) | <0.001 | 0.79 (0.77–0.80) | <0.001 | 0.89 (0.88–0.9) | <0.001 | 1.33 (1.31–1.35) | <0.001 |
| *Mother's BMI before pregnancy* |  |  |  |  |  |  |  |  |
| Mother BMI <18.5 | 1.48 (1.38–1.58) | <0.001 | 0.78 (0.73–0.84) | <0.001 | 1.11 (1.07–1.14) | <0.001 | 1.05 (0.99–1.11) | 0.109 |
| Mother BMI 18.5-24.9 (ref.) | 1.00 |  | 1.00 |  | 1.00 |  | 1.00 |  |
| Mother BMI 25-29.9 | 1.07 (1.04–1.11) | <0.001 | 1.59 (1.55–1.64) | <0.001 | 0.85 (0.84–0.86) | <0.001 | 0.71 (0.69–0.73) | <0.001 |
| Mother BMI 30+ | 1.33 (1.27–1.38) | <0.001 | 2.50 (2.42–2.58) | <0.001 | 0.67 (0.65–0.68) | <0.001 | 0.47 (0.45–0.48) | <0.001 |
| Mother BMI missing | 1.60 (1.50–1.71) | <0.001 | 1.37 (1.30–1.45) | <0.001 | 0.61 (0.59–0.63) | <0.001 | 0.85 (0.81–0.90) | <0.001 |
| *Smoking during pregnancy* |  |  |  |  |  |  |  |  |
| Mother didn't smoke during pregnancy | 1.00 |  | 1.00 |  | 1.00 |  | 1.00 |  |
| Mother smoked during pregnancy | 1.21 (1.17–1.25) | <0.001 | 1.19 (1.16–1.23) | <0.001 | 0.93 (0.92–0.95) | <0.001 | 0.89 (0.87–0.92) | <0.001 |
| Mother smoking missing | 1.88 (1.77–2.01) | <0.001 | 1.25 (1.18–1.33) | <0.001 | 0.95 (0.91–0.99) | 0.006 | 0.76 (0.73–0.80) | <0.001 |
| *Previous induced abortions* |  |  |  |  |  |  |  |  |
| Never had an abortion (ref.) | 1.00 |  | 1.00 |  | 1.00 |  | 1.00 |  |
| Had one abortion | 1.03 (0.99–1.07) | 0.155 | 1.11 (1.07–1.14) | <0.001 | 0.93 (0.91–0.95) | <0.001 | 0.92 (0.89–0.95) | <0.001 |
| Had 2 or more abortions | 1.18 (1.10–1.27) | <0.001 | 1.15 (1.08–1.22) | <0.001 | 0.87 (0.84–0.91) | <0.001 | 0.87 (0.82–0.92) | <0.001 |
| *Previous miscarriages* |  |  |  |  |  |  |  |  |
| Never had a miscarriage (ref.) | 1.00 |  | 1.00 |  | 1.00 |  | 1.00 |  |
| Had one miscarriage | 1.14 (1.10–1.17) | <0.001 | 1.17 (1.14–1.20) | <0.001 | 0.97 (0.95–0.98) | <0.001 | 0.89 (0.87–0.92) | <0.001 |
| Had 2 or more miscarriages | 1.53 (1.45–1.60) | <0.001 | 1.37 (1.31–1.43) | <0.001 | 0.97 (0.94–0.99) | 0.021 | 0.75 (0.72–0.78) | <0.001 |
| *Partner's country of birth (year before childbirth)* | |  |  |  |  |  |  |  |
| Partner born in Finland (ref.) | 1.00 |  | 1.00 |  | 1.00 |  | 1.00 |  |
| Partner born abroad | 0.96 (0.91–1.01) | 0.132 | 0.87 (0.83–0.91) | <0.001 | 1.01 (0.98–1.03) | 0.525 | 1.17 (1.12–1.22) | <0.001 |
| No partner recorded | 1.13 (1.08–1.17) | <0.001 | 1.04 (1.01–1.08) | 0.009 | 0.90 (0.88–0.91) | <0.001 | 0.96 (0.93–0.99) | 0.003 |
| *Mother's relationship status* |  |  |  |  |  |  |  |  |
| Mother married or cohabiting at birth | 1.00 |  | 1.00 |  | 1.00 |  | 1.00 |  |
| Mother not cohabiting or married at birth | 1.14 (1.09–1.20) | <0.001 | 0.99 (0.96–1.03) | 0.744 | 1.05 (1.02–1.07) | <0.001 | 1.05 (1.01–1.08) | 0.010 |
| *Birth year* |  |  |  |  |  |  |  |  |
| 2000 (ref.) | 1.00 |  | 1.00 |  | 1.00 |  | 1.00 |  |
| 2001 | 0.93 (0.87–1.00) | 0.036 | 1.01 (0.95–1.07) | 0.725 | 0.74 (0.72–0.77) | <0.001 | 0.97 (0.92–1.02) | 0.255 |
| 2002 | 0.94 (0.88–1.00) | 0.065 | 0.98 (0.93–1.04) | 0.474 | 0.72 (0.70–0.74) | <0.001 | 0.95 (0.90–1.00) | 0.036 |
| 2003 | 0.90 (0.84–0.96) | 0.002 | 1.01 (0.96–1.07) | 0.637 | 0.63 (0.61–0.65) | <0.001 | 0.95 (0.91–1.00) | 0.046 |
| 2004 | 1.23 (1.14–1.34) | <0.001 | 1.14 (1.06–1.22) | <0.001 | 0.40 (0.39–0.42) | <0.001 | 0.86 (0.81–0.91) | <0.001 |
| 2005 | 1.26 (1.16–1.37) | <0.001 | 1.14 (1.07–1.23) | <0.001 | 0.34 (0.33–0.35) | <0.001 | 0.83 (0.78–0.89) | <0.001 |
| 2006 | 1.38 (1.26–1.51) | <0.001 | 1.20 (1.12–1.30) | <0.001 | 0.31 (0.29–0.32) | <0.001 | 0.79 (0.74–0.84) | <0.001 |
| 2007 | 1.33 (1.21–1.45) | <0.001 | 1.30 (1.20–1.40) | <0.001 | 0.31 (0.30–0.32) | <0.001 | 0.75 (0.70–0.80) | <0.001 |
| 2008 | 1.32 (1.21–1.45) | <0.001 | 1.32 (1.22–1.42) | <0.001 | 0.28 (0.26–0.29) | <0.001 | 0.75 (0.70–0.80) | <0.001 |
| 2009 | 1.29 (1.18–1.42) | <0.001 | 1.25 (1.16–1.35) | <0.001 | 0.25 (0.24–0.26) | <0.001 | 0.80 (0.75–0.86) | <0.001 |
| 2010 | 1.33 (1.21–1.45) | <0.001 | 1.25 (1.16–1.35) | <0.001 | 0.23 (0.22–0.24) | <0.001 | 0.76 (0.71–0.81) | <0.001 |
| 2011 | 1.30 (1.18–1.42) | <0.001 | 1.29 (1.19–1.39) | <0.001 | 0.21 (0.20–0.22) | <0.001 | 0.76 (0.71–0.82) | <0.001 |
| 2012 | 1.31 (1.20–1.44) | <0.001 | 1.29 (1.19–1.39) | <0.001 | 0.20 (0.19–0.21) | <0.001 | 0.75 (0.70–0.80) | <0.001 |
| 2013 | 1.33 (1.21–1.45) | <0.001 | 1.25 (1.16–1.35) | <0.001 | 0.21 (0.20–0.22) | <0.001 | 0.76 (0.71–0.82) | <0.001 |
| 2014 | 1.40 (1.28–1.54) | <0.001 | 1.23 (1.14–1.33) | <0.001 | 0.21 (0.20–0.22) | <0.001 | 0.77 (0.72–0.83) | <0.001 |
| 2015 | 1.34 (1.22–1.47) | <0.001 | 1.14 (1.06–1.24) | 0.001 | 0.18 (0.17–0.19) | <0.001 | 0.81 (0.76–0.87) | <0.001 |
| 2016 | 1.31 (1.19–1.43) | <0.001 | 1.16 (1.08–1.26) | <0.001 | 0.18 (0.17–0.19) | <0.001 | 0.79 (0.74–0.84) | <0.001 |
| 2017 | 1.32 (1.20–1.45) | <0.001 | 1.13 (1.05–1.23) | 0.002 | 0.18 (0.17–0.19) | <0.001 | 0.78 (0.73–0.84) | <0.001 |
| *Hospital district* |  |  |  |  |  |  |  |  |
| Uusimaa (ref.) | 1.00 |  | 1.00 |  | 1.00 |  | 1.00 |  |
| Ahvenanmaa | 0.82 (0.68–1.01) | 0.058 | 1.22 (1.06–1.41) | 0.006 | 0.24 (0.21–0.27) | <0.001 | 0.76 (0.66–0.87) | <0.001 |
| Varsinais-Suomi | 1.24 (1.18–1.30) | <0.001 | 0.75 (0.72–0.79) | <0.001 | 0.49 (0.48–0.51) | <0.001 | 1.10 (1.06–1.15) | <0.001 |
| Satakunta | 1.31 (1.23–1.40) | <0.001 | 0.92 (0.87–0.97) | 0.004 | 1.83 (1.77–1.88) | <0.001 | 1.07 (1.02–1.13) | 0.010 |
| Kanta-Häme | 1.26 (1.17–1.35) | <0.001 | 1.15 (1.08–1.22) | <0.001 | 0.59 (0.56–0.61) | <0.001 | 0.81 (0.76–0.85) | <0.001 |
| Pirkanmaa | 1.07 (1.02–1.12) | 0.007 | 0.70 (0.68–0.73) | <0.001 | 0.70 (1.30–1.36) | <0.001 | 1.34 (1.29–1.39) | <0.001 |
| Päijäthäme | 1.13 (1.05–1.21) | 0.001 | 1.03 (0.98–1.09) | 0.262 | 1.96 (1.91–2.02) | <0.001 | 0.79 (0.75–0.84) | <0.001 |
| Kymenlaakso | 1.06 (0.98–1.15) | 0.149 | 1.18 (1.11–1.25) | <0.001 | 1.36 (1.32–1.41) | <0.001 | 0.98 (0.92–1.04) | 0.455 |
| E-Karjala | 1.13 (1.04–1.24) | 0.006 | 1.10 (1.02–1.18) | 0.009 | 1.10 (1.06–1.15) | <0.001 | 0.89 (0.83–0.95) | 0.001 |
| E-Savo | 1.11 (1.00–1.23) | 0.043 | 1.25 (1.15–1.35) | <0.001 | 1.35 (1.29–1.41) | <0.001 | 0.90 (0.83–0.98) | 0.011 |
| Itä-Savo | 1.08 (0.93–1.27) | 0.314 | 1.60 (1.42–1.80) | <0.001 | 0.75 (0.70–0.81) | <0.001 | 0.57 (0.51–0.64) | <0.001 |
| P-Karjala | 1.02 (0.94–1.10) | 0.681 | 0.63 (0.59–0.68) | <0.001 | 1.37 (1.32–1.42) | <0.001 | 1.14 (1.07–1.22) | <0.001 |
| P-Savo | 1.25 (1.17–1.33) | <0.001 | 0.79 (0.75–0.84) | <0.001 | 0.68 (0.66–0.70) | <0.001 | 1.17 (1.11–1.23) | <0.001 |
| Keski-Suomi | 1.17 (1.10–1.24) | <0.001 | 1.23 (1.17–1.29) | <0.001 | 1.30 (1.26–1.33) | <0.001 | 0.78 (0.74–0.82) | <0.001 |
| E-Pohjanmaa | 0.98 (0.91–1.05) | 0.500 | 1.22 (1.15–1.29) | <0.001 | 2.11 (2.05–2.17) | <0.001 | 0.90 (0.85–0.95) | <0.001 |
| Vaasa | 1.09 (1.01–1.17) | 0.021 | 0.84 (0.79–0.90) | <0.001 | 0.90 (0.87–0.93) | <0.001 | 0.91 (0.86–0.96) | 0.001 |
| K-Pohjanmaa | 1.03 (0.92–1.14) | 0.625 | 1.05 (0.96–1.14) | 0.305 | 0.85 (0.81–0.89) | <0.001 | 0.77 (0.71–0.83) | <0.001 |
| P-Pohjanmaa | 1.15 (1.10–1.21) | <0.001 | 0.92 (0.88–0.96) | <0.001 | 1.12 (1.09–1.14) | <0.001 | 0.94 (0.90–0.97) | 0.001 |
| Kainuu | 1.33 (1.19–1.48) | <0.001 | 1.42 (1.30–1.55) | <0.001 | 1.19 (1.13–1.26) | <0.001 | 0.63 (0.58–0.69) | <0.001 |
| Länsi-Pohja | 1.14 (1.01–1.28) | 0.030 | 0.76 (0.69–0.85) | <0.001 | 1.91 (1.82–2.01) | <0.001 | 1.09 (0.99–1.19) | 0.090 |
| Lappi | 1.12 (1.02–1.22) | 0.019 | 0.92 (0.86–1.00) | 0.042 | 1.00 (0.96–1.04) | 0.877 | 1.10 (1.03–1.19) | 0.007 |
| Standard deviation of random intercept *a_0i_* | 1.72 |  | 1.85 |  | 0.52 |  | 2.50 |  |
| ICC | 0.47 |  | 0.51 |  | 0.08 |  | 0.66 |  |
| Notes: ICC = intra class correlation. * p<0.05; ** p<0.01; ***p<0.001 | | | | | | | | |

Appendix table 4. Likelihood of preterm birth, unplanned C-section, episiotomy or spontaneous vaginal birth by COB for the 10 largest origin countries and women born in Finland, adjusted odds ratios (95% CIs), full results.

|  | **Preterm birth** | | **Unplanned C-section** | | **Episiotomy** | | **Spontaneous vaginal birth** | |
| --- | --- | --- | --- | --- | --- | --- | --- | --- |
|  | *aOR (95% CI)* | *p-value* | *aOR (95% CI)* | *p-value* | *aOR (95% CI)* | *p-value* | *aOR (95% CI)* | *p-value* |
| COUNTRY OF BIRTH (Top 10) |  |  |  |  |  |  |  |  |
| Finland (ref.) | 1.00 |  | 1.00 |  | 1.00 |  | 1.00 |  |
| Sweden | 1.03 (0.93–1.15) | 0.553 | 1.06 (0.97–1.16) | 0.204 | 0.99 (0.94–1.04) | 0.626 | 0.89 (0.82–0.97) | 0.007 |
| FUSSR and Russia | 0.94 (0.85–1.05) | 0.300 | 0.86 (0.78–0.94) | 0.001 | 0.98 (0.93–1.03) | 0.365 | 1.21 (1.12–1.32) | <0.001 |
| Estonia | 0.94 (0.81–1.10) | 0.448 | 0.93 (0.82–1.05) | 0.252 | 1.01 (0.94–1.08) | 0.759 | 1.14 (1.01–1.27) | 0.030 |
| Somalia | 0.96 (0.80–1.15) | 0.660 | 3.49 (3.05–4.00) | <0.001 | 3.24 (2.97–3.53) | <0.001 | 0.32 (0.28–0.37) | <0.001 |
| Iraq | 1.24 (1.00–1.55) | 0.049 | 1.67 (1.39–2.02) | <0.001 | 1.33 (1.19–1.49) | <0.001 | 0.47 (0.39–0.56) | <0.001 |
| Former Yugoslavia | 0.85 (0.67–1.09) | 0.207 | 0.90 (0.72–1.11) | 0.309 | 1.27 (1.14–1.42) | <0.001 | 0.97 (0.80–1.17) | 0.732 |
| Thailand | 1.22 (0.98–1.51) | 0.071 | 1.92 (1.63–2.28) | <0.001 | 1.01 (0.91–1.12) | 0.886 | 0.51 (0.43–0.60) | <0.001 |
| China | 1.03 (0.80–1.33) | 0.826 | 1.08 (0.88–1.32) | 0.454 | 1.42 (1.28–1.58) | <0.001 | 0.79 (0.66–0.96) | 0.018 |
| Vietnam | 1.30 (1.00–1.68) | 0.048 | 1.36 (1.09–1.71) | 0.007 | 1.38 (1.22–1.56) | <0.001 | 0.76 (0.62–0.95) | 0.014 |
| Turkey | 1.07 (0.79–1.45) | 0.663 | 1.37 (1.07–1.75) | 0.014 | 1.18 (1.02–1.36) | 0.024 | 0.65 (0.51–0.82) | <0.001 |
| HOUSEHOLD INCOME QUINTILES |  |  |  |  |  |  |  |  |
| Poorest (ref.) | 1.00 |  | 1.00 |  | 1.00 |  | 1.00 |  |
| Poor | 0.98 (0.94–1.02) | 0.298 | 1.00 (0.97–1.04) | 0.836 | 1.02 (1.00–1.04) | 0.110 | 0.95 (0.92–0.98) | 0.001 |
| Middle | 0.91 (0.87–0.95) | <0.001 | 1.00 (0.97–1.04) | 0.864 | 1.07 (1.04–1.09) | <0.001 | 0.93 (0.90–0.96) | <0.001 |
| Richer | 0.91 (0.87–0.95) | <0.001 | 1.01 (0.97–1.05) | 0.572 | 1.12 (1.10–1.14) | <0.001 | 0.90 (0.87–0.93) | <0.001 |
| Richest | 0.92 (0.88–0.97) | 0.001 | 0.96 (0.93–1.00) | 0.067 | 1.12 (1.09–1.15) | <0.001 | 0.90 (0.87–0.94) | <0.001 |
| CONTROL VARIABLES |  |  |  |  |  |  |  |  |
| *Mother's age at birth* |  |  |  |  |  |  |  |  |
| Mother aged <20 at birth | 0.88 (0.82–0.95) | 0.002 | 0.44 (0.42–0.48) | <0.001 | 0.98 (0.95–1.02) | 0.334 | 3.25 (3.07–3.45) | <0.001 |
| Mother aged 20-24 at birth | 0.89 (0.86–0.93) | <0.001 | 0.68 (0.66–0.71) | <0.001 | 0.96 (0.94–0.98) | <0.001 | 1.84 (1.79–1.89) | <0.001 |
| Mother aged 25-29 at birth (ref.) | 1.00 |  | 1.00 |  | 1.00 |  | 1.00 |  |
| Mother aged 30-34 at birth | 1.10 (1.07–1.14) | <0.001 | 1.48 (1.45–1.52) | <0.001 | 1.05 (1.04–1.07) | <0.001 | 0.55 (0.54–0.56) | <0.001 |
| Mother aged 35+ at birth | 1.46 (1.41–1.51) | <0.001 | 2.34 (2.27–2.41) | <0.001 | 1.12 (1.10–1.14) | <0.001 | 0.28 (0.27–0.29) | <0.001 |
| *Number of previous births* |  |  |  |  |  |  |  |  |
| Parity 0 (ref.) | 1.00 |  | 1.00 |  | 1.00 |  | 1.00 |  |
| Parity 1 | 0.57 (0.55–0.59) | <0.001 | 0.25 (0.25–0.26) | <0.001 | 0.19 (0.19–0.19) | <0.001 | 7.02 (6.87–7.17) | <0.001 |
| Parity 2 | 0.56 (0.54–0.59) | <0.001 | 0.13 (0.12–0.13) | <0.001 | 0.06 (0.05–0.06) | <0.001 | 11.8 (11.4–12.1) | <0.001 |
| Parity 3+ | 0.66 (0.63–0.69) | <0.001 | 0.10 (0.10–0.11) | <0.001 | 0.02 (0.02–0.02) | <0.001 | 17.8 (17.1–18.6) | <0.001 |
| *Child sex* |  |  |  |  |  |  |  |  |
| Child sex: boy (ref.) | 1.00 |  | 1.00 |  | 1.00 |  | 1.00 |  |
| Child sex: girl | 0.80 (0.78–0.82) | <0.001 | 0.79 (0.77–0.80) | <0.001 | 0.89 (0.88–0.90) | <0.001 | 1.33 (1.31–1.35) | <0.001 |
| *Mother's BMI before pregnancy* |  |  |  |  |  |  |  |  |
| Mother BMI <18.5 | 1.49 (1.39–1.59) | <0.001 | 0.77 (0.72–0.83) | <0.001 | 1.11 (1.07–1.15) | <0.001 | 1.05 (0.99–1.11) | 0.120 |
| Mother BMI 18.5-24.9 (ref.) | 1.00 |  | 1.00 |  | 1.00 |  | 1.00 |  |
| Mother BMI 25-29.9 | 1.07 (1.03–1.10) | <0.001 | 1.59 (1.55–1.64) | <0.001 | 0.85 (0.83–0.86) | <0.001 | 0.71 (0.70–0.73) | <0.001 |
| Mother BMI 30+ | 1.32 (1.26–1.37) | <0.001 | 2.50 (2.42–2.58) | <0.001 | 0.67 (0.65–0.69) | <0.001 | 0.47 (0.46–0.49) | <0.001 |
| Mother BMI missing | 1.62 (1.52–1.72) | <0.001 | 1.38 (1.31–1.46) | <0.001 | 0.61 (0.59–0.63) | <0.001 | 0.85 (0.81–0.89) | <0.001 |
| *Smoking during pregnancy* |  |  |  |  |  |  |  |  |
| Mother didn't smoke during pregnancy | 1.00 |  | 1.00 |  | 1.00 |  | 1.00 |  |
| Mother smoked during pregnancy | 1.21 (1.17–1.25) | <0.001 | 1.20 (1.17–1.23) | <0.001 | 0.93 (0.92–0.95) | <0.001 | 0.89 (0.87–0.91) | <0.001 |
| Mother smoking missing | 1.89 (1.78–2.02) | <0.001 | 1.26 (1.18–1.34) | <0.001 | 0.95 (0.91–0.99) | 0.007 | 0.76 (0.72–0.80) | <0.001 |
| *Previous induced abortions* |  |  |  |  |  |  |  |  |
| Never had an abortion (ref.) | 1.00 |  | 1.00 |  | 1.00 |  | 1.00 |  |
| Had one abortion | 1.03 (0.99–1.07) | 0.178 | 1.11 (1.08–1.15) | <0.001 | 0.93 (0.91–0.95) | <0.001 | 0.92 (0.89–0.94) | <0.001 |
| Had 2 or more abortions | 1.19 (1.10–1.28) | <0.001 | 1.16 (1.09–1.24) | <0.001 | 0.88 (0.85–0.92) | <0.001 | 0.85 (0.80–0.90) | <0.001 |
| *Previous miscarriages* |  |  |  |  |  |  |  |  |
| Never had a miscarriage (ref.) | 1.00 |  | 1.00 |  | 1.00 |  | 1.00 |  |
| Had one miscarriage | 1.13 (1.10–1.17) | <0.001 | 1.17 (1.13–1.20) | <0.001 | 0.97 (0.95–0.99) | <0.001 | 0.89 (0.87–0.92) | <0.001 |
| Had 2 or more miscarriages | 1.52 (1.45–1.60) | <0.001 | 1.38 (1.32–1.44) | <0.001 | 0.96 (0.94–0.99) | 0.020 | 0.75 (0.72–0.78) | <0.001 |
| *Partner's country of birth (year before childbirth)* | |  |  |  |  |  |  |  |
| Partner born in Finland (ref.) | 1.00 |  | 1.00 |  | 1.00 |  | 1.00 |  |
| Partner born abroad | 0.95 (0.90–1.01) | 0.120 | 0.88 (0.83–0.92) | <0.001 | 0.97 (0.94–1.00) | 0.044 | 1.17 (1.12–1.23) | <0.001 |
| No partner recorded | 1.12 (1.08–1.17) | <0.001 | 1.05 (1.01–1.08) | 0.007 | 0.89 (0.88–0.91) | <0.001 | 0.95 (0.92–0.98) | 0.001 |
| *Mother's relationship status* |  |  |  |  |  |  |  |  |
| Mother married or cohabiting at birth | 1.00 |  | 1.00 |  | 1.00 |  | 1.00 |  |
| Mother not cohabiting or married at birth | 1.14 (1.09–1.19) | <0.001 | 0.99 (0.95–1.03) | 0.598 | 1.05 (1.03–1.07) | <0.001 | 1.05 (1.01–1.08) | 0.009 |
| *Birth year* |  |  |  |  |  |  |  |  |
| 2000 (ref.) | 1.00 |  | 1.00 |  | 1.00 |  | 1.00 |  |
| 2001 | 0.94 (0.88–1.01) | 0.070 | 1.01 (0.95–1.06) | 0.862 | 0.74 (0.72–0.77) | <0.001 | 0.97 (0.93–1.02) | 0.266 |
| 2002 | 0.94 (0.88–1.01) | 0.094 | 0.98 (0.92–1.04) | 0.453 | 0.72 (0.70–0.75) | <0.001 | 0.95 (0.91–1.00) | 0.042 |
| 2003 | 0.91 (0.85–0.97) | 0.006 | 1.01 (0.96–1.07) | 0.629 | 0.63 (0.61–0.65) | <0.001 | 0.95 (0.91–1.00) | 0.052 |
| 2004 | 1.26 (1.16–1.37) | <0.001 | 1.14 (1.06–1.22) | <0.001 | 0.40 (0.39–0.42) | <0.001 | 0.86 (0.81–0.91) | <0.001 |
| 2005 | 1.29 (1.18–1.40) | <0.001 | 1.15 (1.07–1.23) | <0.001 | 0.34 (0.32–0.35) | <0.001 | 0.83 (0.78–0.88) | <0.001 |
| 2006 | 1.41 (1.28–1.54) | <0.001 | 1.21 (1.12–1.30) | <0.001 | 0.31 (0.29–0.32) | <0.001 | 0.78 (0.73–0.84) | <0.001 |
| 2007 | 1.36 (1.24–1.49) | <0.001 | 1.30 (1.21–1.41) | <0.001 | 0.31 (0.29–0.32) | <0.001 | 0.74 (0.70–0.80) | <0.001 |
| 2008 | 1.33 (1.22–1.46) | <0.001 | 1.32 (1.22–1.43) | <0.001 | 0.28 (0.26–0.29) | <0.001 | 0.74 (0.70–0.80) | <0.001 |
| 2009 | 1.32 (1.20–1.45) | <0.001 | 1.26 (1.17–1.36) | <0.001 | 0.24 (0.23–0.26) | <0.001 | 0.80 (0.75–0.85) | <0.001 |
| 2010 | 1.35 (1.23–1.48) | <0.001 | 1.26 (1.17–1.36) | <0.001 | 0.23 (0.22–0.24) | <0.001 | 0.76 (0.71–0.81) | <0.001 |
| 2011 | 1.33 (1.21–1.46) | <0.001 | 1.28 (1.19–1.38) | <0.001 | 0.21 (0.20–0.22) | <0.001 | 0.76 (0.71–0.82) | <0.001 |
| 2012 | 1.33 (1.22–1.46) | <0.001 | 1.30 (1.20–1.40) | <0.001 | 0.20 (0.19–0.21) | <0.001 | 0.75 (0.70–0.80) | <0.001 |
| 2013 | 1.34 (1.22–1.47) | <0.001 | 1.27 (1.17–1.37) | <0.001 | 0.21 (0.20–0.22) | <0.001 | 0.76 (0.71–0.81) | <0.001 |
| 2014 | 1.43 (1.30–1.57) | <0.001 | 1.23 (1.14–1.33) | <0.001 | 0.20 (0.20–0.21) | <0.001 | 0.77 (0.72–0.83) | <0.001 |
| 2015 | 1.37 (1.25–1.51) | <0.001 | 1.14 (1.06–1.24) | 0.001 | 0.18 (0.17–0.19) | <0.001 | 0.81 (0.76–0.87) | <0.001 |
| 2016 | 1.34 (1.22–1.47) | <0.001 | 1.18 (1.09–1.27) | <0.001 | 0.18 (0.17–0.19) | <0.001 | 0.78 (0.73–0.84) | <0.001 |
| 2017 | 1.34 (1.22–1.48) | <0.001 | 1.13 (1.05–1.23) | 0.002 | 0.18 (0.17–0.18) | <0.001 | 0.78 (0.73–0.84) | <0.001 |
| *Hospital district* |  |  |  |  |  |  |  |  |
| Uusimaa (ref.) | 1.00 |  | 1.00 |  | 1.00 |  | 1.00 |  |
| Ahvenanmaa | 0.83 (0.67–1.01) | 0.067 | 1.16 (1.00–1.34) | 0.052 | 0.24 (0.21–0.27) | <0.001 | 0.78 (0.67–0.90) | 0.001 |
| Varsinais-Suomi | 1.24 (1.18–1.30) | <0.001 | 0.75 (0.72–0.79) | <0.001 | 0.50 (0.48–0.51) | <0.001 | 1.11 (1.06–1.15) | <0.001 |
| Satakunta | 1.32 (1.23–1.40) | <0.001 | 0.92 (0.87–0.97) | 0.003 | 1.84 (1.79–1.90) | <0.001 | 1.08 (1.02–1.14) | 0.007 |
| Kanta-Häme | 1.26 (1.17–1.35) | <0.001 | 1.14 (1.07–1.21) | <0.001 | 0.59 (0.57–0.61) | <0.001 | 0.81 (0.77–0.86) | <0.001 |
| Pirkanmaa | 1.06 (1.01–1.11) | 0.012 | 0.70 (0.68–0.73) | <0.001 | 1.33 (1.31–1.36) | <0.001 | 1.34 (1.29–1.39) | <0.001 |
| Päijäthäme | 1.13 (1.05–1.21) | 0.001 | 1.03 (0.97–1.09) | 0.281 | 1.98 (1.92–2.04) | <0.001 | 0.79 (0.75–0.84) | <0.001 |
| Kymenlaakso | 1.07 (0.99–1.16) | 0.107 | 1.18 (1.10–1.25) | <0.001 | 1.38 (1.33–1.43) | <0.001 | 0.97 (0.91–1.03) | 0.305 |
| E-Karjala | 1.14 (1.04–1.24) | 0.005 | 1.11 (1.03–1.19) | 0.006 | 1.11 (1.07–1.16) | <0.001 | 0.88 (0.82–0.94) | <0.001 |
| E-Savo | 1.12 (1.01–1.24) | 0.035 | 1.24 (1.15–1.35) | <0.001 | 1.36 (1.30–1.42) | <0.001 | 0.91 (0.84–0.98) | 0.014 |
| Itä-Savo | 1.08 (0.92–1.27) | 0.347 | 1.60 (1.42–1.80) | <0.001 | 0.76 (0.70–0.82) | <0.001 | 0.57 (0.50–0.64) | <0.001 |
| P-Karjala | 1.02 (0.94–1.11) | 0.629 | 0.64 (0.60–0.69) | <0.001 | 1.37 (1.32–1.42) | <0.001 | 1.13 (1.07–1.21) | <0.001 |
| P-Savo | 1.25 (1.17–1.33) | <0.001 | 0.79 (0.75–0.84) | <0.001 | 0.68 (0.66–0.71) | <0.001 | 1.16 (1.10–1.22) | <0.001 |
| Keski-Suomi | 1.17 (1.10–1.24) | <0.001 | 1.23 (1.17–1.29) | <0.001 | 1.31 (1.27–1.35) | <0.001 | 0.78 (0.75–0.82) | <0.001 |
| E-Pohjanmaa | 0.97 (0.90–1.04) | 0.417 | 1.21 (1.15–1.28) | <0.001 | 2.13 (2.07–2.19) | <0.001 | 0.91 (0.86–0.96) | 0.001 |
| Vaasa | 1.09 (1.01–1.18) | 0.020 | 0.83 (0.78–0.88) | <0.001 | 0.90 (0.87–0.93) | <0.001 | 0.92 (0.87–0.98) | 0.008 |
| K-Pohjanmaa | 1.03 (0.93–1.14) | 0.575 | 1.05 (0.96–1.14) | 0.300 | 0.85 (0.81–0.90) | <0.001 | 0.77 (0.71–0.83) | <0.001 |
| P-Pohjanmaa | 1.15 (1.10–1.21) | <0.001 | 0.92 (0.88–0.96) | <0.001 | 1.12 (1.10–1.15) | <0.001 | 0.94 (0.90–0.98) | 0.001 |
| Kainuu | 1.33 (1.19–1.48) | <0.001 | 1.41 (1.29–1.54) | <0.001 | 1.19 (1.13–1.26) | <0.001 | 0.64 (0.59–0.70) | <0.001 |
| Länsi-Pohja | 1.14 (1.02–1.29) | 0.027 | 0.76 (0.68–0.85) | <0.001 | 1.94 (1.84–2.04) | <0.001 | 1.09 (0.99–1.20) | 0.067 |
| Lappi | 1.12 (1.02–1.23) | 0.018 | 0.93 (0.86–1.00) | 0.055 | 1.00 (0.96–1.04) | 0.969 | 1.10 (1.02–1.19) | 0.009 |
| Standard deviation of random intercept *a_0i_* | 1.72 |  | 1.85 |  | 0.52 |  | 2.49 |  |
| ICC | 0.47 |  | 0.51 |  | 0.08 |  | 0.65 |  |
| Notes: ICC = intra class correlation. * p<0.05; ** p<0.01; ***p<0.001 | | | | | | | | |

Appendix table 5. Likelihood of preterm birth, unplanned C-section, episiotomy or spontaneous vaginal birth by income level of COB and household income, with interaction between COB income level and household income, adjusted odds ratios (95% CIs), full results.

|  | **Preterm birth** | | **Unplanned C-section** | | **Episiotomy** | | **Spontaneous vaginal birth** | |
| --- | --- | --- | --- | --- | --- | --- | --- | --- |
|  | *aOR (95% CI)* | *p-value* | *aOR (95% CI)* | *p-value* | *aOR (95% CI)* | *p-value* | *aOR (95% CI)* | *p-value* |
| COUNTRY OF BIRTH’S INCOME LEVEL |  |  |  |  |  |  |  |  |
| Finland i.e. non-migrant (ref.) | 1.00 |  | 1.00 |  | 1.00 |  | 1.00 |  |
| Low income country | 1.06 (0.90–1.24) | 0.508 | 3.30 (2.92–3.74) | <0.001 | 2.36 (2.17–2.56) | 0.000 | 0,33 (0,29–0,37) | 0.000 |
| Lower middle income | 1.39 (1.16–1.66) | <0.001 | 2.68 (2.33–3.09) | <0.001 | 1.19 (1.08–1.31) | 0.000 | 0,42 (0,36–0,48) | 0.000 |
| Upper middle income | 1.02 (0.91–1.14) | 0.739 | 1.21 (1.10–1.32) | <0.001 | 1.12 (1.06–1.18) | 0.000 | 0,77 (0,71–0,84) | 0.000 |
| High income country | 0.99 (0.86–1.14) | 0.914 | 0.99 (0.88–1.13) | 0.927 | 0.98 (0.91–1.06) | 0.637 | 1,05 (0,94–1,18) | 0.380 |
| HOUSEHOLD INCOME QUINTILES |  |  |  |  |  |  |  |  |
| Poorest (ref.) | 1.00 |  | 1.00 |  | 1.00 |  | 1.00 |  |
| Poor | 0.98 (0.94–1.02) | 0.287 | 1.00 (0.97–1.04) | 0.818 | 1.02 (1.00–1.04) | 0.102 | 0,95 (0,92–0,98) | 0.002 |
| Middle | 0.91 (0.87–0.95) | <0.001 | 1.01 (0.97–1.05) | 0.753 | 1.07 (1.04–1.09) | 0.000 | 0,93 (0,90–0,96) | 0.000 |
| Richer | 0.91 (0.87–0.95) | <0.001 | 1.01 (0.97–1.05) | 0.569 | 1.13 (1.10–1.15) | 0.000 | 0,90 (0,88–0,94) | 0.000 |
| Richest | 0.94 (0.89–0.98) | 0.007 | 0.97 (0.93–1.01) | 0.106 | 1.12 (1.10–1.15) | 0.000 | 0,91 (0,87–0,94) | 0.000 |
| *Interaction HH income quintiles*COB income level* |  |  |  |  |  |  |  |  |
| Finland/poorest (ref.) | 1.00 |  | 1.00 |  | 1.00 |  | 1.00 |  |
| Low income*poorer | 1.49 (1.13–1.96) | 0.004 | 0.98 (0.79–1.22) | 0.855 | 0.83 (0.71–0.97) | 0.017 | 1,09 (0,89–1,34) | 0.397 |
| Low income*middle | 0.80 (0.48–1.31) | 0.369 | 0.96 (0.70–1.32) | 0.793 | 0.77 (0.61–0.95) | 0.017 | 0,98 (0,72–1,34) | 0.901 |
| Low income*richer | 1.48 (0.79–2.78) | 0.225 | 1.26 (0.80–1.98) | 0.323 | 0.55 (0.40–0.76) | 0.000 | 0,76 (0,48–1,21) | 0.244 |
| Low income*richest | 0.61 (0.21–1.74) | 0.352 | 0.46 (0.24–0.89) | 0.022 | 0.75 (0.51–1.11) | 0.150 | 1,11 (0,60–2,06) | 0.734 |
| Lower middle income*poorer | 1.17 (0.90–1.54) | 0.241 | 0.95 (0.77–1.18) | 0.655 | 0.94 (0.81–1.09) | 0.408 | 1,02 (0,83–1,25) | 0.846 |
| Lower middle income*middle | 0.92 (0.67–1.28) | 0.632 | 0.80 (0.63–1.03) | 0.082 | 1.05 (0.89–1.24) | 0.543 | 0,93 (0,74–1,18) | 0.565 |
| Lower middle income*richer | 0.81 (0.56–1.18) | 0.281 | 0.90 (0.69–1.17) | 0.412 | 0.93 (0.78–1.11) | 0.396 | 0,92 (0,71–1,19) | 0.513 |
| Lower middle income*richest | 0.99 (0.66–1.49) | 0.968 | 1.00 (0.74–1.34) | 0.995 | 0.92 (0.75–1.11) | 0.367 | 0,79 (0,58–1,06) | 0.120 |
| Upper middle income*poorer | 0.97 (0.82–1.16) | 0.743 | 1.05 (0.92–1.21) | 0.465 | 0.97 (0.89–1.06) | 0.549 | 1,06 (0,93–1,19) | 0.395 |
| Upper middle income*middle | 1.01 (0.83–1.24) | 0.889 | 0.87 (0.74–1.02) | 0.081 | 0.95 (0.87–1.05) | 0.319 | 1,16 (1,00–1,33) | 0.044 |
| Upper middle income*richer | 1.01 (0.82–1.25) | 0.934 | 0.88 (0.74–1.04) | 0.141 | 0.89 (0.81–0.98) | 0.021 | 1,21 (1,04–1,41) | 0.013 |
| Upper middle income*richest | 0.91 (0.72–1.13) | 0.390 | 0.83 (0.70–0.99) | 0.035 | 0.93 (0.84–1.02) | 0.123 | 1,15 (0,98–1,35) | 0.083 |
| High income*poorer | 0.95 (0.77–1.16) | 0.598 | 0.93 (0.77–1.11) | 0.407 | 1.02 (0.92–1.14) | 0.688 | 0,99 (0,85–1,16) | 0.917 |
| High income*middle | 1.09 (0.88–1.34) | 0.441 | 0.99 (0.83–1.19) | 0.924 | 1.02 (0.92–1.14) | 0.700 | 1,05 (0,90–1,23) | 0.532 |
| High income*richer | 1.01 (0.82–1.25) | 0.895 | 1.06 (0.89–1.25) | 0.544 | 0.94 (0.85–1.05) | 0.279 | 0,98 (0,84–1,15) | 0.828 |
| High income*richest | 0.77 (0.62–0.96) | 0.021 | 0.86 (0.72–1.02) | 0.087 | 1.07 (0.97–1.18) | 0.173 | 0,97 (0,83–1,13) | 0.682 |
| CONTROL VARIABLES |  |  |  |  |  |  |  |  |
| *Mother's age at birth* |  |  |  |  |  |  |  |  |
| Mother aged <20 at birth | 0.89 (0.83–0.96) | 0.003 | 0.44 (0.41–0.47) | <0.001 | 0.98 (0.95–1.02) | 0.363 | 3,25 (3,07–3,45) | 0.000 |
| Mother aged 20-24 at birth | 0.90 (0.86–0.93) | <0.001 | 0.68 (0.66–0.70) | <0.001 | 0.96 (0.94–0.98) | 0.000 | 1,83 (1,79–1,89) | 0.000 |
| Mother aged 25-29 at birth (ref.) | 1.00 |  | 1.00 |  | 1.00 |  | 1.00 |  |
| Mother aged 30-34 at birth | 1.10 (1.07–1.14) | <0.001 | 1.48 (1.45–1.52) | <0.001 | 1.05 (1.03–1.06) | 0.000 | 0,55 (0,54–0,56) | 0.000 |
| Mother aged 35+ at birth | 1.46 (1.41–1.51) | <0.001 | 2.35 (2.28–2.42) | <0.001 | 1.11 (1.09–1.13) | 0.000 | 0,28 (0,27–0,29) | 0.000 |
| *Number of previous births* |  |  |  |  |  |  |  |  |
| Parity 0 (ref.) | 1.00 |  | 1.00 |  | 1.00 |  | 1.00 |  |
| Parity 1 | 0.58 (0.56–0.59) | <0.001 | 0.25 (0.25–0.26) | <0.001 | 0.19 (0.19–0.19) | 0.000 | 7,02 (6,88–7,17) | 0.000 |
| Parity 2 | 0.57 (0.55–0.59) | <0.001 | 0.13 (0.12–0.13) | <0.001 | 0.06 (0.05–0.06) | 0.000 | 11,8 (11,4–12,1) | 0.000 |
| Parity 3+ | 0.66 (0.63–0.69) | <0.001 | 0.11 (0.10–0.11) | <0.001 | 0.02 (0.02–0.02) | 0.000 | 17,8 (17,1–18,6) | 0.000 |
| *Child sex* |  |  |  |  |  |  |  |  |
| Child sex: boy (ref.) | 1.00 |  | 1.00 |  | 1.00 |  | 1.00 |  |
| Child sex: girl | 0.80 (0.78–0.82) | <0.001 | 0.79 (0.77–0.80) | <0.001 | 0.89 (0.88–0.90) | 0.000 | 1,33 (1,31–1,35) | 0.000 |
| *Mother's BMI before pregnancy* |  |  |  |  |  |  |  |  |
| Mother BMI <18.5 | 1.48 (1.38–1.58) | <0.001 | 0.78 (0.73–0.84) | <0.001 | 1.11 (1.07–1.15) | 0.000 | 1,05 (0,99–1,11) | 0.114 |
| Mother BMI 18.5-24.9 (ref.) | 1.00 |  | 1.00 |  | 1.00 |  | 1.00 |  |
| Mother BMI 25-29.9 | 1.07 (1.04–1.11) | <0.001 | 1.59 (1.55–1.64) | <0.001 | 0.85 (0.84–0.86) | 0.000 | 0,71 (0,69–0,73) | 0.000 |
| Mother BMI 30+ | 1.33 (1.27–1.38) | <0.001 | 2.50 (2.42–2.58) | <0.001 | 0.67 (0.65–0.68) | 0.000 | 0,47 (0,45–0,48) | 0.000 |
| Mother BMI missing | 1.60 (1.5–1.71) | <0.001 | 1.37 (1.30–1.45) | <0.001 | 0.61 (0.59–0.63) | 0.000 | 0,85 (0,81–0,90) | 0.000 |
| *Smoking during pregnancy* |  |  |  |  |  |  |  |  |
| Mother didn't smoke during pregnancy | 1.00 |  | 1.00 |  | 1.00 |  | 1.00 |  |
| Mother smoked during pregnancy | 1.21 (1.17–1.25) | <0.001 | 1.20 (1.16–1.23) | <0.001 | 0.93 (0.92–0.95) | 0.000 | 0,89 (0,87–0,92) | 0.000 |
| Mother smoking missing | 1.88 (1.77–2.01) | <0.001 | 1.25 (1.18–1.33) | <0.001 | 0.95 (0.91–0.99) | 0.007 | 0,76 (0,73–0,80) | 0.000 |
| *Previous induced abortions* |  |  |  |  |  |  |  |  |
| Never had an abortion (ref.) | 1.00 |  | 1.00 |  | 1.00 |  | 1.00 |  |
| Had one abortion | 1.03 (0.99–1.07) | 0.151 | 1.11 (1.07–1.15) | <0.001 | 0.93 (0.91–0.95) | 0.000 | 0,92 (0,89–0,95) | 0.000 |
| Had 2 or more abortions | 1.18 (1.10–1.26) | <0.001 | 1.15 (1.08–1.22) | <0.001 | 0.87 (0.84–0.91) | 0.000 | 0,87 (0,82–0,92) | 0.000 |
| *Previous miscarriages* |  |  |  |  |  |  |  |  |
| Never had a miscarriage (ref.) | 1.00 |  | 1.00 |  | 1.00 |  | 1.00 |  |
| Had one miscarriage | 1.14 (1.10–1.17) | <0.001 | 1.17 (1.14–1.20) | <0.001 | 0.97 (0.95–0.98) | 0.000 | 0,89 (0,87–0,92) | 0.000 |
| Had 2 or more miscarriages | 1.53 (1.45–1.60) | <0.001 | 1.37 (1.31–1.43) | <0.001 | 0.97 (0.94–0.99) | 0.022 | 0,75 (0,72–0,78) | 0.000 |
| *Partner's country of birth (year before childbirth)* | |  |  |  |  |  |  |  |
| Partner born in Finland (ref.) | 1.00 |  | 1.00 |  | 1.00 |  | 1.00 |  |
| Partner born abroad | 0.95 (0.90–1.01) | 0.098 | 0.87 (0.83–0.91) | <0.001 | 1.01 (0.98–1.03) | 0.589 | 1,17 (1,12–1,22) | 0.000 |
| No partner recorded | 1.13 (1.08–1.17) | <0.001 | 1.04 (1.01–1.08) | 0.008 | 0.90 (0.88–0.92) | 0.000 | 0,96 (0,93–0,98) | 0.003 |
| *Mother's relationship status* |  |  |  |  |  |  |  |  |
| Mother married or cohabiting at birth | 1.00 |  | 1.00 |  | 1.00 |  | 1.00 |  |
| Mother not cohabiting or married at birth | 1.14 (1.09–1.20) | <0.001 | 0.99 (0.96–1.03) | 0.761 | 1.05 (1.02–1.07) | 0.000 | 1,05 (1,01–1,08) | 0.011 |
| *Birth year* |  |  |  |  |  |  |  |  |
| 2000 (ref.) | 1.00 |  | 1.00 |  | 1.00 |  | 1.00 |  |
| 2001 | 0.93 (0.87–1.00) | 0.036 | 1.01 (0.95–1.07) | 0.729 | 0.74 (0.72–0.77) | 0.000 | 0,97 (0,92–1,02) | 0.256 |
| 2002 | 0.94 (0.88–1.00) | 0.066 | 0.98 (0.93–1.04) | 0.471 | 0.72 (0.70–0.74) | 0.000 | 0,95 (0,90–1,00) | 0.036 |
| 2003 | 0.90 (0.84–0.96) | 0.003 | 1.01 (0.96–1.07) | 0.644 | 0.63 (0.61–0.65) | 0.000 | 0,95 (0,91–1,00) | 0.046 |
| 2004 | 1.23 (1.14–1.34) | <0.001 | 1.14 (1.06–1.22) | <0.001 | 0.40 (0.39–0.42) | 0.000 | 0,86 (0,81–0,92) | 0.000 |
| 2005 | 1.26 (1.16–1.37) | <0.001 | 1.14 (1.06–1.23) | <0.001 | 0.34 (0.33–0.35) | 0.000 | 0,83 (0,78–0,89) | 0.000 |
| 2006 | 1.38 (1.26–1.51) | <0.001 | 1.20 (1.11–1.30) | <0.001 | 0.31 (0.29–0.32) | 0.000 | 0,79 (0,74–0,84) | 0.000 |
| 2007 | 1.33 (1.21–1.45) | <0.001 | 1.30 (1.20–1.40) | <0.001 | 0.31 (0.30–0.32) | 0.000 | 0,75 (0,70–0,80) | 0.000 |
| 2008 | 1.32 (1.21–1.45) | <0.001 | 1.32 (1.22–1.42) | <0.001 | 0.28 (0.26–0.29) | 0.000 | 0,75 (0,70–0,80) | 0.000 |
| 2009 | 1.29 (1.18–1.42) | <0.001 | 1.25 (1.16–1.35) | <0.001 | 0.25 (0.23–0.26) | 0.000 | 0,80 (0,75–0,86) | 0.000 |
| 2010 | 1.33 (1.21–1.45) | <0.001 | 1.25 (1.16–1.35) | <0.001 | 0.23 (0.22–0.24) | 0.000 | 0,76 (0,71–0,81) | 0.000 |
| 2011 | 1.30 (1.18–1.42) | <0.001 | 1.29 (1.19–1.39) | <0.001 | 0.21 (0.20–0.22) | 0.000 | 0,76 (0,71–0,82) | 0.000 |
| 2012 | 1.31 (1.20–1.44) | <0.001 | 1.29 (1.19–1.39) | <0.001 | 0.20 (0.19–0.21) | 0.000 | 0,75 (0,70–0,80) | 0.000 |
| 2013 | 1.33 (1.21–1.46) | <0.001 | 1.25 (1.16–1.35) | <0.001 | 0.21 (0.20–0.22) | 0.000 | 0,76 (0,71–0,82) | 0.000 |
| 2014 | 1.40 (1.28–1.54) | <0.001 | 1.23 (1.14–1.33) | <0.001 | 0.21 (0.20–0.22) | 0.000 | 0,77 (0,72–0,83) | 0.000 |
| 2015 | 1.34 (1.22–1.47) | <0.001 | 1.14 (1.06–1.24) | 0.001 | 0.18 (0.17–0.19) | 0.000 | 0,81 (0,76–0,87) | 0.000 |
| 2016 | 1.31 (1.19–1.43) | <0.001 | 1.16 (1.08–1.26) | <0.001 | 0.18 (0.17–0.19) | 0.000 | 0,79 (0,74–0,84) | 0.000 |
| 2017 | 1.32 (1.20–1.45) | <0.001 | 1.13 (1.05–1.23) | 0.002 | 0.18 (0.17–0.19) | 0.000 | 0,78 (0,73–0,84) | 0.000 |
| *Hospital district* |  |  |  |  |  |  |  |  |
| Uusimaa (ref.) | 1.00 |  | 1.00 |  | 1.00 |  | 1.00 |  |
| Ahvenanmaa | 0.83 (0.68–1.01) | 0.062 | 0.00 (1.06–1.41) | 0.005 | 0.24 (0.21–0.27) | 0.000 | 0,76 (0,66–0,88) | 0.000 |
| Varsinais-Suomi | 1.24 (1.18–1.30) | <0.001 | 0.75 (0.72–0.79) | <0.001 | 0.49 (0.48–0.51) | 0.000 | 1,10 (1,06–1,15) | 0.000 |
| Satakunta | 1.31 (1.23–1.40) | <0.001 | 0.92 (0.87–0.97) | 0.004 | 1.83 (1.77–1.88) | 0.000 | 1,07 (1,02–1,13) | 0.009 |
| Kanta-Häme | 1.26 (1.17–1.35) | <0.001 | 1.15 (1.08–1.22) | <0.001 | 0.59 (0.56–0.61) | 0.000 | 0,81 (0,76–0,85) | 0.000 |
| Pirkanmaa | 1.07 (1.02–1.12) | 0.007 | 0.70 (0.68–0.73) | <0.001 | 1.33 (1.30–1.35) | 0.000 | 1,34 (1,29–1,39) | 0.000 |
| Päijäthäme | 1.13 (1.06–1.21) | 0.001 | 1.03 (0.98–1.09) | 0.264 | 1.96 (1.90–2.02) | 0.000 | 0,79 (0,75–0,84) | 0.000 |
| Kymenlaakso | 1.06 (0.98–1.15) | 0.143 | 1.18 (1.10–1.25) | <0.001 | 1.36 (1.31–1.41) | 0.000 | 0,98 (0,92–1,04) | 0.471 |
| E-Karjala | 1.13 (1.04–1.24) | 0.006 | 1.10 (1.02–1.18) | 0.010 | 1.10 (1.06–1.14) | 0.000 | 0,89 (0,83–0,95) | 0.001 |
| E-Savo | 1.11 (1.00–1.23) | 0.041 | 1.25 (1.15–1.35) | <0.001 | 1.35 (1.29–1.41) | 0.000 | 0,90 (0,83–0,98) | 0.011 |
| Itä-Savo | 1.09 (0.93–1.27) | 0.309 | 1.60 (1.42–1.80) | <0.001 | 0.75 (0.70–0.81) | 0.000 | 0,57 (0,51–0,64) | 0.000 |
| P-Karjala | 1.02 (0.94–1.10) | 0.657 | 0.64 (0.59–0.68) | <0.001 | 1.37 (1.32–1.42) | 0.000 | 1,14 (1,07–1,22) | 0.000 |
| P-Savo | 1.25 (1.18–1.33) | <0.001 | 0.79 (0.75–0.84) | <0.001 | 0.68 (0.66–0.70) | 0.000 | 1,17 (1,11–1,23) | 0.000 |
| Keski-Suomi | 1.17 (1.10–1.24) | <0.001 | 1.23 (1.17–1.30) | <0.001 | 1.30 (1.26–1.33) | 0.000 | 0,78 (0,74–0,82) | 0.000 |
| E-Pohjanmaa | 0.98 (0.91–1.05) | 0.513 | 1.22 (1.15–1.29) | <0.001 | 2.11 (2.05–2.17) | 0.000 | 0,90 (0,85–0,95) | 0.000 |
| Vaasa | 1.09 (1.01–1.17) | 0.019 | 0.84 (0.79–0.89) | <0.001 | 0.90 (0.87–0.93) | 0.000 | 0,91 (0,86–0,96) | 0.001 |
| K-Pohjanmaa | 1.03 (0.93–1.14) | 0.612 | 1.05 (0.96–1.14) | 0.303 | 0.85 (0.81–0.89) | 0.000 | 0,77 (0,71–0,83) | 0.000 |
| P-Pohjanmaa | 1.16 (1.10–1.21) | <0.001 | 0.92 (0.88–0.96) | <0.001 | 1.12 (1.09–1.14) | 0.000 | 0,94 (0,90–0,97) | 0.001 |
| Kainuu | 1.33 (1.19–1.48) | <0.001 | 1.42 (1.30–1.55) | <0.001 | 1.19 (1.13–1.25) | 0.000 | 0,64 (0,58–0,69) | 0.000 |
| Länsi-Pohja | 1.14 (1.01–1.28) | 0.030 | 0.76 (0.69–0.85) | <0.001 | 1.91 (1.82–2.02) | 0.000 | 1,09 (0,99–1,19) | 0.093 |
| Lappi | 1.12 (1.02–1.22) | 0.018 | 0.92 (0.86–1.00) | 0.044 | 1.00 (0.96–1.04) | 0.895 | 1,10 (1,03–1,19) | 0.008 |
| Standard deviation of random intercept *a_0i_* | 1.72 |  | 1.85 |  | 0.52 |  | 2.50 |  |
| ICC | 0.47 |  | 0.51 |  | 0.08 |  | 0.66 |  |
| Notes: ICC = intra class correlation. * p<0.05; ** p<0.01; ***p<0.001 | | | | | | | | |

Appendix table 6. Likelihood of preterm birth, unplanned C-section, episiotomy or spontaneous vaginal birth by time lived in Finland adjusted odds ratios (95% CIs).

|  | **Preterm birth** | | **Unplanned C-section** | | **Episiotomy** | | **Spontaneous vaginal birth** | |
| --- | --- | --- | --- | --- | --- | --- | --- | --- |
|  | *aOR (95% CI)* | *p-value* | *aOR (95% CI)* | *p-value* | *aOR (95% CI)* | *p-value* | *aOR (95% CI)* | *p-value* |
| HOUSEHOLD INCOME QUINTILES |  |  |  |  |  |  |  |  |
| Poorest (ref.) | 1.00 |  | 1.00 |  | 1.00 |  | 1.00 |  |
| Poor | 0.98 (0.94–1.02) | 0.357 | 1.00 (0.97–1.04) | 0.987 | 1.01 (0.99–1.03) | 0.305 | 0.96 (0.93–0.98) | 0.003 |
| Middle | 0.91 (0.87–0.95) | <0.001 | 0.99 (0.96–1.03) | 0.754 | 1.06 (1.04–1.08) | <0.001 | 0.94 (0.91–0.97) | <0.001 |
| Richer | 0.91 (0.87–0.95) | <0.001 | 1.00 (0.97–1.04) | 0.818 | 1.11 (1.09–1.14) | <0.001 | 0.91 (0.88–0.94) | <0.001 |
| Richest | 0.93 (0.88–0.97) | 0.001 | 0.95 (0.92–0.99) | 0.018 | 1.12 (1.09–1.14) | <0.001 | 0.91 (0.88–0.94) | <0.001 |
| COB INCOME LEVEL & TIME SINCE MIGRATION |  |  |  |  |  |  |  |  |
| Born in Finland (ref.) | 1.00 |  | 1.00 |  | 1.00 |  | 1.00 |  |
| Low income country, <2 years | 1.17 (0.82–1.65) | 0.385 | 4.48 (3.53–5.69) | <0.001 | 1.75 (1.47–2.07) | <0.001 | 0.30 (0.23–0.38) | <0.001 |
| Lower middle income, <2 years | 1.13 (0.87–1.46) | 0.374 | 2.27 (1.89–2.72) | <0.001 | 1.23 (1.09–1.38) | <0.001 | 0.43 (0.35–0.51) | <0.001 |
| Upper middle income, <2 years | 0.92 (0.77–1.10) | 0.369 | 1.28 (1.12–1.46) | <0.001 | 1.09 (1.02–1.18) | 0.018 | 0.80 (0.71–0.90) | <0.001 |
| High income country, <2 years | 0.81 (0.62–1.04) | 0.103 | 0.88 (0.72–1.08) | 0.217 | 0.97 (0.87–1.09) | 0.617 | 1.12 (0.94–1.34) | 0.204 |
| Low income country, 2-5 years | 0.94 (0.76–1.17) | 0.575 | 3.13 (2.69–3.66) | <0.001 | 2.12 (1.92–2.35) | <0.001 | 0.31 (0.27–0.36) | <0.001 |
| Lower middle income, 2-5 years | 1.33 (1.12–1.59) | 0.001 | 2.92 (2.57–3.32) | <0.001 | 1.12 (1.03–1.23) | 0.009 | 0.38 (0.33–0.43) | <0.001 |
| Upper middle income, 2-5 years | 1.04 (0.94–1.17) | 0.438 | 1.20 (1.10–1.32) | <0.001 | 1.11 (1.05–1.17) | <0.001 | 0.77 (0.71–0.83) | <0.001 |
| High income country, 2-5 years | 0.85 (0.73–0.99) | 0.041 | 0.92 (0.81–1.04) | 0.163 | 1.00 (0.93–1.07) | 0.934 | 1.15 (1.03–1.28) | 0.013 |
| Low income country, 6-9 years | 1.15 (0.91–1.45) | 0.236 | 3.41 (2.86–4.06) | <0.001 | 2.30 (2.03–2.62) | <0.001 | 0.33 (0.28–0.39) | <0.001 |
| Lower middle income, 6-9 years | 1.49 (1.18–1.88) | 0.001 | 2.38 (1.98–2.86) | <0.001 | 1.27 (1.11–1.45) | 0.001 | 0.40 (0.34–0.48) | <0.001 |
| Upper middle income, 6-9 years | 0.99 (0.86–1.14) | 0.915 | 1.03 (0.92–1.16) | 0.580 | 1.04 (0.97–1.12) | 0.279 | 0.85 (0.77–0.94) | 0.001 |
| High income country, 6-9 years | 0.91 (0.74–1.12) | 0.374 | 0.91 (0.77–1.08) | 0.277 | 0.97 (0.88–1.07) | 0.489 | 1.27 (1.10–1.46) | 0.001 |
| Low income country, 10 or more years | 1.35 (1.09–1.68) | 0.007 | 2.70 (2.26–3.22) | <0.001 | 2.19 (1.93–2.50) | <0.001 | 0.38 (0.32–0.45) | <0.001 |
| Lower middle income, 10 or more years | 1.63 (1.29–2.06) | <0.001 | 2.06 (1.68–2.52) | <0.001 | 1.05 (0.91–1.21) | 0.468 | 0.47 (0.39–0.57) | <0.001 |
| Upper middle income, 10 or more years | 0.97 (0.86–1.10) | 0.669 | 1.06 (0.96–1.18) | 0.251 | 1.03 (0.97–1.10) | 0.308 | 0.93 (0.85–1.02) | 0.134 |
| High income country, 10 or more years | 1.01 (0.92–1.11) | 0.768 | 0.99 (0.92–1.07) | 0.827 | 1.00 (0.96–1.05) | 0.954 | 0.96 (0.89–1.04) | 0.310 |
| CONTROL VARIABLES |  |  |  |  |  |  |  |  |
| *Mother's age at birth* |  |  |  |  |  |  |  |  |
| Mother aged <20 at birth | 0.89 (0.82–0.96) | 0.003 | 0.44 (0.41–0.47) | <0.001 | 0.98 (0.95–1.02) | 0.322 | 3.25 (3.07–3.45) | <0.001 |
| Mother aged 20-24 at birth | 0.90 (0.86–0.93) | <0.001 | 0.68 (0.66–0.70) | <0.001 | 0.96 (0.94–0.97) | <0.001 | 1.84 (1.79–1.89) | <0.001 |
| Mother aged 25-29 at birth (ref.) | 1.00 |  | 1.00 |  | 1.00 |  | 1.00 |  |
| Mother aged 30-34 at birth | 1.10 (1.07–1.14) | <0.001 | 1.49 (1.45–1.52) | <0.001 | 1.05 (1.03–1.06) | <0.001 | 0.55 (0.54–0.56) | <0.001 |
| Mother aged 35+ at birth | 1.46 (1.41–1.51) | <0.001 | 2.35 (2.28–2.42) | <0.001 | 1.11 (1.09–1.13) | <0.001 | 0.28 (0.27–0.29) | <0.001 |
| *Number of previous births* |  |  |  |  |  |  |  |  |
| Parity 0 (ref.) | 1.00 |  | 1.00 |  | 1.00 |  | 1.00 |  |
| Parity 1 | 0.57 (0.56–0.59) | <0.001 | 0.25 (0.25–0.26) | <0.001 | 0.19 (0.19–0.19) | <0.001 | 7.02 (6.88–7.17) | <0.001 |
| Parity 2 | 0.57 (0.55–0.59) | <0.001 | 0.13 (0.12–0.13) | <0.001 | 0.06 (0.05–0.06) | <0.001 | 11.7 (11.4–12.1) | <0.001 |
| Parity 3+ | 0.65 (0.62–0.69) | <0.001 | 0.11 (0.10–0.11) | <0.001 | 0.02 (0.02–0.02) | <0.001 | 17.8 (17.1–18.5) | <0.001 |
| *Child sex* |  |  |  |  |  |  |  |  |
| Child sex: boy (ref.) | 1.00 |  | 1.00 |  | 1.00 |  | 1.00 |  |
| Child sex: girl | 0.80 (0.78–0.82) | <0.001 | 0.79 (0.77–0.80) | <0.001 | 0.89 (0.88–0.90) | <0.001 | 1.33 (1.31–1.35) | <0.001 |
| *Mother's BMI before pregnancy* |  |  |  |  |  |  |  |  |
| Mother BMI <18.5 | 1.48 (1.38–1.58) | <0.001 | 0.78 (0.73–0.84) | <0.001 | 1.11 (1.07–1.14) | <0.001 | 1.05 (0.99–1.11) | 0.112 |
| Mother BMI 18.5-24.9 (ref.) | 1.00 |  | 1.00 |  | 1.00 |  | 1.00 |  |
| Mother BMI 25-29.9 | 1.07 (1.04–1.11) | <0.001 | 1.59 (1.55–1.64) | <0.001 | 0.85 (0.84–0.86) | <0.001 | 0.71 (0.69–0.73) | <0.001 |
| Mother BMI 30+ | 1.32 (1.27–1.38) | <0.001 | 2.50 (2.42–2.58) | <0.001 | 0.67 (0.65–0.68) | <0.001 | 0.47 (0.45–0.48) | <0.001 |
| Mother BMI missing | 1.60 (1.50–1.71) | <0.001 | 1.37 (1.30–1.45) | <0.001 | 0.61 (0.59–0.63) | <0.001 | 0.85 (0.81–0.90) | <0.001 |
| *Smoking during pregnancy* |  |  |  |  |  |  |  |  |
| Mother didn't smoke during pregnancy | 1.00 |  | 1.00 |  | 1.00 |  | 1.00 |  |
| Mother smoked during pregnancy | 1.21 (1.17–1.25) | <0.001 | 1.20 (1.16–1.23) | <0.001 | 0.93 (0.92–0.95) | <0.001 | 0.89 (0.87–0.92) | <0.001 |
| Mother smoking missing | 1.88 (1.77–2.01) | <0.001 | 1.25 (1.18–1.33) | <0.001 | 0.95 (0.91–0.99) | 0.007 | 0.76 (0.73–0.80) | <0.001 |
| *Previous induced abortions* |  |  |  |  |  |  |  |  |
| Never had an abortion (ref.) | 1.00 |  | 1.00 |  | 1.00 |  | 1.00 |  |
| Had one abortion | 1.03 (0.99–1.07) | 0.168 | 1.11 (1.07–1.15) | <0.001 | 0.93 (0.91–0.95) | <0.001 | 0.92 (0.89–0.95) | <0.001 |
| Had 2 or more abortions | 1.18 (1.10–1.26) | <0.001 | 1.15 (1.08–1.23) | <0.001 | 0.87 (0.84–0.91) | <0.001 | 0.87 (0.82–0.92) | <0.001 |
| *Previous miscarriages* |  |  |  |  |  |  |  |  |
| Never had a miscarriage (ref.) | 1.00 |  | 1.00 |  | 1.00 |  | 1.00 |  |
| Had one miscarriage | 1.14 (1.10–1.17) | <0.001 | 1.17 (1.14–1.20) | <0.001 | 0.97 (0.95–0.98) | <0.001 | 0.89 (0.87–0.92) | <0.001 |
| Had 2 or more miscarriages | 1.53 (1.45–1.60) | <0.001 | 1.37 (1.31–1.43) | <0.001 | 0.97 (0.94–0.99) | 0.021 | 0.75 (0.72–0.78) | <0.001 |
| *Partner's country of birth (year before childbirth)* | |  |  |  |  |  |  |  |
| Partner born in Finland (ref.) | 1.00 |  | 1.00 |  | 1.00 |  | 1.00 |  |
| Partner born abroad | 0.97 (0.91–1.02) | 0.245 | 0.87 (0.83–0.91) | <0.001 | 1.01 (0.98–1.03) | 0.530 | 1.16 (1.12–1.21) | <0.001 |
| No partner recorded | 1.13 (1.08–1.17) | <0.001 | 1.05 (1.01–1.08) | 0.006 | 0.90 (0.88–0.91) | <0.001 | 0.96 (0.93–0.98) | 0.002 |
| *Mother's relationship status* |  |  |  |  |  |  |  |  |
| Mother married or cohabiting at birth | 1.00 |  | 1.00 |  | 1.00 |  | 1.00 |  |
| Mother not cohabiting or married at birth | 1.14 (1.09–1.19) | <0.001 | 0.99 (0.96–1.03) | 0.784 | 1.05 (1.02–1.07) | <0.001 | 1.05 (1.01–1.08) | 0.011 |
| *Birth year* |  |  |  |  |  |  |  |  |
| 2000 (ref.) | 1.00 |  | 1.00 |  | 1.00 |  | 1.00 |  |
| 2001 | 0.93 (0.87–1.00) | 0.036 | 1.01 (0.95–1.07) | 0.718 | 0.74 (0.72–0.77) | <0.001 | 0.97 (0.92–1.02) | 0.249 |
| 2002 | 0.94 (0.88–1.00) | 0.064 | 0.98 (0.93–1.04) | 0.474 | 0.72 (0.70–0.74) | <0.001 | 0.95 (0.90–1.00) | 0.034 |
| 2003 | 0.90 (0.84–0.96) | 0.002 | 1.01 (0.96–1.07) | 0.633 | 0.63 (0.61–0.65) | <0.001 | 0.95 (0.91–1.00) | 0.044 |
| 2004 | 1.23 (1.13–1.34) | <0.001 | 1.14 (1.06–1.22) | <0.001 | 0.40 (0.39–0.42) | <0.001 | 0.86 (0.81–0.91) | <0.001 |
| 2005 | 1.26 (1.16–1.37) | <0.001 | 1.14 (1.07–1.23) | <0.001 | 0.34 (0.33–0.35) | <0.001 | 0.83 (0.78–0.89) | <0.001 |
| 2006 | 1.38 (1.26–1.51) | <0.001 | 1.20 (1.12–1.30) | <0.001 | 0.31 (0.29–0.32) | <0.001 | 0.79 (0.74–0.84) | <0.001 |
| 2007 | 1.33 (1.21–1.45) | <0.001 | 1.30 (1.20–1.40) | <0.001 | 0.31 (0.30–0.32) | <0.001 | 0.75 (0.70–0.80) | <0.001 |
| 2008 | 1.32 (1.21–1.45) | <0.001 | 1.32 (1.22–1.42) | <0.001 | 0.28 (0.26–0.29) | <0.001 | 0.75 (0.70–0.80) | <0.001 |
| 2009 | 1.29 (1.18–1.42) | <0.001 | 1.25 (1.16–1.35) | <0.001 | 0.25 (0.24–0.26) | <0.001 | 0.80 (0.75–0.86) | <0.001 |
| 2010 | 1.33 (1.21–1.45) | <0.001 | 1.25 (1.16–1.35) | <0.001 | 0.23 (0.22–0.24) | <0.001 | 0.76 (0.71–0.81) | <0.001 |
| 2011 | 1.30 (1.18–1.42) | <0.001 | 1.29 (1.19–1.39) | <0.001 | 0.21 (0.20–0.22) | <0.001 | 0.76 (0.71–0.81) | <0.001 |
| 2012 | 1.31 (1.20–1.44) | <0.001 | 1.29 (1.20–1.39) | <0.001 | 0.20 (0.19–0.21) | <0.001 | 0.75 (0.70–0.80) | <0.001 |
| 2013 | 1.33 (1.21–1.46) | <0.001 | 1.26 (1.16–1.36) | <0.001 | 0.21 (0.20–0.22) | <0.001 | 0.76 (0.71–0.81) | <0.001 |
| 2014 | 1.40 (1.28–1.54) | <0.001 | 1.23 (1.14–1.33) | <0.001 | 0.21 (0.20–0.22) | <0.001 | 0.77 (0.72–0.83) | <0.001 |
| 2015 | 1.34 (1.22–1.47) | <0.001 | 1.15 (1.06–1.24) | 0.001 | 0.18 (0.17–0.19) | <0.001 | 0.81 (0.75–0.86) | <0.001 |
| 2016 | 1.31 (1.19–1.43) | <0.001 | 1.17 (1.08–1.26) | <0.001 | 0.18 (0.17–0.19) | <0.001 | 0.79 (0.73–0.84) | <0.001 |
| 2017 | 1.32 (1.20–1.45) | <0.001 | 1.14 (1.05–1.23) | 0.001 | 0.18 (0.17–0.19) | <0.001 | 0.78 (0.73–0.84) | <0.001 |
| *Hospital district* |  |  |  |  |  |  |  |  |
| Uusimaa (ref.) | 1.00 |  | 1.00 |  | 1.00 |  | 1.00 |  |
| Ahvenanmaa | 0.83 (0.68–1.01) | 0.061 | 1.22 (1.06–1.41) | 0.006 | 0.24 (0.21–0.27) | <0.001 | 0.76 (0.66–0.87) | <0.001 |
| Varsinais-Suomi | 1.24 (1.18–1.30) | <0.001 | 0.75 (0.72–0.79) | <0.001 | 0.49 (0.48–0.51) | <0.001 | 1.10 (1.06–1.15) | <0.001 |
| Satakunta | 1.31 (1.23–1.40) | <0.001 | 0.92 (0.87–0.97) | 0.003 | 1.83 (1.77–1.88) | <0.001 | 1.08 (1.02–1.13) | 0.008 |
| Kanta-Häme | 1.26 (1.17–1.35) | <0.001 | 1.14 (1.08–1.21) | <0.001 | 0.59 (0.56–0.61) | <0.001 | 0.81 (0.76–0.86) | <0.001 |
| Pirkanmaa | 1.07 (1.02–1.12) | 0.007 | 0.70 (0.67–0.73) | <0.001 | 1.33 (1.30–1.36) | <0.001 | 1.34 (1.29–1.39) | <0.001 |
| Päijäthäme | 1.13 (1.05–1.21) | 0.001 | 1.03 (0.97–1.09) | 0.286 | 1.96 (1.91–2.02) | <0.001 | 0.79 (0.75–0.84) | <0.001 |
| Kymenlaakso | 1.06 (0.98–1.15) | 0.146 | 1.17 (1.10–1.25) | <0.001 | 1.36 (1.32–1.41) | <0.001 | 0.98 (0.92–1.04) | 0.514 |
| E-Karjala | 1.13 (1.04–1.24) | 0.006 | 1.10 (1.02–1.18) | 0.011 | 1.10 (1.06–1.15) | <0.001 | 0.89 (0.83–0.95) | 0.001 |
| E-Savo | 1.11 (1.00–1.23) | 0.042 | 1.24 (1.15–1.35) | <0.001 | 1.35 (1.29–1.41) | <0.001 | 0.90 (0.84–0.98) | 0.012 |
| Itä-Savo | 1.08 (0.93–1.27) | 0.316 | 1.60 (1.42–1.80) | <0.001 | 0.75 (0.70–0.81) | <0.001 | 0.57 (0.51–0.64) | <0.001 |
| P-Karjala | 1.02 (0.94–1.00) | 0.676 | 0.63 (0.59–0.68) | <0.001 | 1.37 (1.32–1.42) | <0.001 | 1.15 (1.08–1.22) | <0.001 |
| P-Savo | 1.25 (1.17–1.33) | <0.001 | 0.79 (0.75–0.83) | <0.001 | 0.68 (0.66–0.70) | <0.001 | 1.17 (1.11–1.23) | <0.001 |
| Keski-Suomi | 1.17 (1.10–1.24) | <0.001 | 1.23 (1.17–1.29) | <0.001 | 1.30 (1.26–1.33) | <0.001 | 0.78 (0.74–0.82) | <0.001 |
| E-Pohjanmaa | 0.98 (0.91–1.05) | 0.494 | 1.22 (1.15–1.29) | <0.001 | 2.11 (2.05–2.17) | <0.001 | 0.90 (0.86–0.95) | <0.001 |
| Vaasa | 1.09 (1.01–1.17) | 0.021 | 0.84 (0.79–0.89) | <0.001 | 0.90 (0.87–0.93) | <0.001 | 0.91 (0.86–0.96) | 0.001 |
| K-Pohjanmaa | 1.03 (0.92–1.14) | 0.637 | 1.04 (0.96–1.14) | 0.330 | 0.85 (0.81–0.89) | <0.001 | 0.77 (0.71–0.84) | <0.001 |
| P-Pohjanmaa | 1.15 (1.10–1.21) | <0.001 | 0.92 (0.88–0.96) | <0.001 | 1.12 (1.09–1.14) | <0.001 | 0.94 (0.90–0.98) | 0.001 |
| Kainuu | 1.33 (1.19–1.48) | <0.001 | 1.41 (1.29–1.55) | <0.001 | 1.19 (1.13–1.26) | <0.001 | 0.64 (0.58–0.69) | <0.001 |
| Länsi-Pohja | 1.14 (1.01–1.28) | 0.033 | 0.76 (0.69–0.85) | <0.001 | 1.91 (1.82–2.01) | <0.001 | 1.09 (0.99–1.20) | 0.073 |
| Lappi | 1.11 (1.02–1.22) | 0.021 | 0.92 (0.85–0.99) | 0.036 | 1.00 (0.95–1.04) | 0.870 | 1.11 (1.03–1.19) | 0.005 |
| Standard deviation of random intercept *a_0i_* | 1.73 |  | 1.85 |  | 0.52 |  | 2.50 |  |
| ICC | 0.47 |  | 0.51 |  | 0.08 |  | 0.66 |  |
| Notes: ICC = intra class correlation. * p<0.05; ** p<0.01; ***p<0.001 | | | | | | | | |
